# Predictive performance of cardiometabolic indices for prediabetes progression to diabetes and reversion to normoglycemia in adults: a cohort study

**DOI:** 10.3389/fnut.2026.1784247

**Published:** 2026-05-14

**Authors:** Yilin Zhu, Yangxuan He, Shusheng Fang, Hong Chen, Jingshan Jiang, Yunxiang Ming, Song Leng

**Affiliations:** 1 Health Management Center, the Second Affiliated Hospital of Dalian Medical University, Dalian, China; 2 School of Public Health, Dalian Medical University, Dalian, China; 3 Department of Gastroenterology, the Second Affiliated Hospital of Dalian Medical University, Dalian, China

**Keywords:** cardiometabolic index, cohort study, diabetes, prediabetes, triglycerides-glucose

## Abstract

**Background:**

To evaluate the associations of cardiometabolic indices with prediabetes progression and reversal and to identify noninvasive markers with relatively better discriminatory performance for risk stratification.

**Methods:**

We analyzed 35,525 adults (aged ≥18 years) with prediabetes from the Dalian Health Management Cohort. Associations of cardiometabolic indices with prediabetes progression and reversal were assessed using Cox proportional hazards models and restricted cubic splines (RCS). Relative discriminatory performance was compared using time-dependent receiver operating characteristic (ROC) curves, and mediation and subgroup analyses were subsequently performed.

**Results:**

Over 90,810.98 person-years, 3,125 (8.8%) participants progressed to diabetes, and 13,605 (38.8%) reverted to normoglycemia. After multivariable adjustment and standardization, triglyceride glucose-waist height ratio (TyG-WHtR) exhibited the strongest associations among the evaluated indicators (progression: HR = 2.15, 95% CI: 2.06–2.24; reversal: HR = 0.60, 95% CI: 0.59–0.62) and relatively better discriminatory performance, as supported by time-dependent ROC analysis and the calculation of C-index, NRI, and IDI. Mediation analysis indicated that gamma-glutamyl transpeptidase (GGT) and neutrophil count accounted for modest proportions of the associations with prediabetes progression (8.26 and 3.88%, respectively) and reversal (4.05 and 0.80%, respectively) through potential indirect pathways.

**Conclusion:**

Cardiometabolic indices are associated with prediabetes progression to diabetes and reversal to normoglycemia, with TyG-WHtR showing relatively better discriminatory performance in this cohort. Inflammation and oxidative stress may represent potential pathways underlying the aforementioned associations.

## 1 Introduction

Prediabetes, characterized by elevated glycemic levels below diabetes thresholds, is a prevalent metabolic disorder linked to aging and a high-risk state for type 2 diabetes (T2DM) (1, 2). Globally, an estimated 352 million people have prediabetes, with projections reaching 587 million by 2045. In China, a 2018 survey reported a prediabetes prevalence of 38.1% (3, 4). Prediabetes is driven by multiple factors and increases the risk of cardiovascular disease (CVD) and other complications (5). In particular, the substantial burden of atherosclerotic cardiovascular disease (ASCVD) and the persistent challenges in maintaining care quality underscore the urgent need for optimized management strategies in high-risk individuals (6). Individuals with prediabetes may progress to diabetes, remain in a prediabetic state, or revert to normal fasting glucose (NFG) (7). Annually, 5–10% of individuals with prediabetes progress to diabetes, often with elevated CVD risk (8). Therefore, identifying factors influencing prediabetes progression and reversal is critical.

Cardiometabolic risk factors are pivotal in prediabetes dynamics. Individuals with diabetes often exhibit metabolic abnormalities, including hypertension, dyslipidemia, obesity, and insulin resistance (IR), which independently elevate CVD risk and are linked to diabetes progression (9). IR, inflammation, and metabolic disturbances are strongly associated with diabetes risk (10). Several cardiometabolic indices, such as cardiometabolic index (CMI), atherogenic index of plasma (AIP), triglyceride-glucose index (TyG), triglyceride glucose-body mass index (TyG-BMI), triglyceride glucose-waist circumference (TyG-WC), and TyG-WHtR, serve as accessible IR biomarkers (11–13). Although these indices are associated with various diseases, their specific roles in prediabetes progression and reversal remain underexplored, with varying predictive performance (14–17). This study evaluates the associations of 12 cardiometabolic indices with prediabetes progression and reversal in a large cohort to identify the most effective index.

## 2 Methods

### 2.1 Study population

Participants were drawn from the Dalian Health Management Cohort (DHMC; Cohort number: CCC2023112102), established in 2014 by the Second Affiliated Hospital of Dalian Medical University to study population health and inform prevention strategies. The detailed description of this cohort can be found elsewhere (17, 18). We included 44,500 adults (aged ≥18 years) with ≥2 health examinations between 2014 and 2024. Exclusion criteria included baseline diabetes, normoglycemia, or hypoglycemia; missing data for cardiometabolic indices; absent baseline fasting plasma glucose (FPG) or HbA1c; or with other major diseases or malignancies. After exclusions, 35,525 participants remained (Supplementary Figure 1). Baseline was defined as the initial health examination. Follow-up continued until the primary endpoint was identified (either progression to diabetes or reversion to normoglycemia) or until the last visit for those lost to follow-up or remaining in a prediabetic state, whichever occurred first. The event time was assigned as the date of the follow-up examination at which progression to diabetes or reversion to normoglycemia was first detected. The study adhered to the Declaration of Helsinki and was approved by the Ethics Review Committee of the Second Affiliated Hospital of Dalian Medical University (Approval No. KY2025-603-01).

### 2.2 Data collection and measurement

Anthropometric measurements included height and weight (measured in light clothing without shoes) and waist circumference (WC, measured 1 cm above the navel). Laboratory assessments involved fasting venous blood samples (after ≥8 h of fasting) analyzed for hemoglobin, FPG, total cholesterol (TC), triglycerides (TG), high-density lipoprotein cholesterol (HDL-C), and low-density lipoprotein cholesterol (LDL-C) using an automated biochemical immunoassay system. BMI was calculated as weight (kg) divided by height squared (m^2^). WHtR was calculated as WC (cm) divided by height (cm).

### 2.3 Assessment of cardiometabolic indices and blood biomarkers

The study evaluated 12 cardiometabolic indices: BMI, CMI, AIP, lipid accumulation product (LAP), weight-adjusted waist index (WWI), VAI, WHtR, body roundness index (BRI), TyG, TyG-BMI, TyG-WC, and TyG-WHtR. The calculation formulas for these indicators were provided in Supplementary Table 1.

In addition, candidate blood biomarkers were identified through rigorous laboratory assessment. Candidate mediators were selected based on the following considerations: prior evidence supporting relevance to dysglycemia progression (19); plausible biological links to inflammation, oxidative stress, or metabolic dysregulation; and acceptable completeness and quality in the database. These biomarkers included systematic inflammation markers: white blood cell (WBC), neutrophil count, lymphocyte count and monocyte count; oxidative stress markers: GGT and uric acid. Additional details are provided in Supplementary Table 2.

### 2.4 Definition of prediabetes, diabetes, and normoglycemia

FPG was measured using an automatic biochemical analyzer (Hitachi 7150). Prediabetes, diabetes, and normoglycemia were classified per American Diabetes Association criteria. Prediabetes was defined as FPG 5.6–6.9 mmol/L (100–124 mg/dL) or HbA1c 5.7–6.4% (20). Diabetes was defined as FPG ≥ 7.0 mmol/L (126 mg/dL), HbA1c ≥ 6.5%, or self-reported medical history (20). Normoglycemia was defined as FPG < 5.6 mmol/L (100 mg/dL) (20). Participants were grouped by prediabetes status changes within 1–5 years: progression to diabetes, persistent prediabetes, or reversion to normoglycemia.

### 2.5 Covariates

Demographic data, including sex, medical history, medication use, smoking, and alcohol consumption, were collected via structured questionnaires. Medication use was defined as any self-reported current medication use at baseline. Because the original medication records were heterogeneous and incomplete, this variable may include glucose-lowering drugs, lipid-lowering drugs, antihypertensive agents, GLP-1 receptor agonists, and other medications with potential metabolic effects; therefore, medication use was analyzed as a binary variable (yes/no). Smoking status was categorized as “never smoked” or “current smoker” based on the question, “Do you currently smoke (21)?” Alcohol consumption was defined as drinking at least once a week in the past year and further classified as excessively (≥210 g/wk for males and 140 g/wk for females), occasionally (0–210 g/wk for males and 0–140 g/wk for females), or never (21). Blood pressure was measured after ≥5 min of rest using an electronic sphygmomanometer. Hypertension was defined as systolic blood pressure (SBP) ≥ 140 mmHg, diastolic blood pressure (DBP) ≥ 90 mmHg, use of antihypertensive medications, or self-reported hypertension (22). Dyslipidemia was defined as TG > 1.70 mmol/L (150 mg/dL), TC > 5.18 mmol/L (200 mg/dL), LDL-C > 3.37 mmol/L (130 mg/dL), and/or HDL-C ≤ 1.04 mmol/L (40 mg/dL) (23).

### 2.6 Statistical analysis

Continuous variables with non-normal distributions were reported as medians (interquartile ranges); those with normal distributions were reported as means ± standard deviations. Group differences were tested using one-way ANOVA or Kruskal-Wallis H tests, as appropriate. Categorical variables were reported as counts (percentages) and compared using chi-square tests. Missing values of covariates were shown in Supplementary Figure 2. Missing covariates were imputed using random forest imputation. Dose–response relationships were examined using RCS. Cox proportional hazards models assessed associations of the 12 indices with progression to diabetes and reversion to normoglycemia. Model 1 was unadjusted; Model 2 adjusted for age and sex; Model 3 further adjusted for dyslipidemia, hypertension, medication use, drinking, and smoking. The proportional hazards assumption was verified using Schoenfeld residuals. Confounders were selected based on demographic characteristics, prior literature, clinical considerations, change-in-estimate criteria, and directed acyclic graph analysis (Supplementary Figure 3). Time-dependent ROC curves, time-dependent Harrell’s concordance indices (C-index), net reclassification improvement (NRI), and integrated discrimination improvement (IDI) were used to assess relative discriminatory performance. Formal calibration analysis was not performed because the primary aim of this study was comparative assessment of indices rather than development of a full clinical prediction model. In the mediation analysis, cardiometabolic indices with relatively better discriminatory performance for both outcomes was selected as the exposure variable. Drawing upon previous research (19, 24), we constructed two models to determine whether the selected biomarkers might serve as potential mediators. A linear regression model was employed to assess the association between the exposure variable and the selected biomarkers, while a multivariable-adjusted Cox proportional hazards model was used to examine the relationship between the biomarkers and the progression to or reversal of prediabetes. Biomarkers exhibiting statistical significance in both models with consistent effect directions were identified as potential mediators. Mediation analysis was conducted using the “mediation” package in R, calculating the proportion of mediation and obtaining 95% confidence intervals via non-parametric bootstrapping (1,000 resamples). Subgroup analyses stratified by age, sex, BMI, hypertension, dyslipidemia, baseline FPG, and medication use were performed for the top-performing indicator. Insulin resistance index-based stratification was not performed because HOMA-IR and related measures were unavailable in the database. To evaluate robustness, we also conducted three sensitivity analyses: redefinition of prediabetes using the World Health Organization (WHO) criteria [FPG 6.1–6.9 mmol/L (110–125 mg/dL)] (25); exclusion of participants lost to follow-up or with follow-up duration <2 years; and exclusion of participants with any self-reported current medication use at baseline.

All analyses were performed in R 4.4.3, with two-sided *p* < 0.05 indicating significance.

## 3 Results

### 3.1 Characteristics of participants

Among 35,525 participants, 21,114 (59.4%) were male. During the median follow-up of 1.99 years, 3,125 (8.8%) participants progressed to diabetes, 13,605 (38.3%) reverted to normoglycemia and 18,795 participants (52.9%) remained in the prediabetes state. Compared with those persistent prediabetes, participants progression to diabetes were more likely to be male and older, had higher levels of WC, SBP, DBP, Hb, FPG, TC, LDL-C and TG, lower levels of HDL-C, and had a higher proportions of smoking, drinking, hypertension, medication use and dyslipidemia (overall *p* < 0.001; Table 1).

**Table 1:** Baseline characteristics of the study population.

Variable	Reversion to normoglycemia (*n* = 13,605)	Persistent prediabete (*n* = 18,795)	Progression to diabetes (*n* = 3,125)	*p*-value
Age, years	40.00 (32.00, 50.00)	48.00 (38.00, 57.00)	52.00 (44.00, 60.00)	<0.001
SBP, mmHg	124.00 (114.00, 134.00)	131.00 (120.00, 141.00)	136.00 (126.00, 149.00)	<0.001
DBP, mmHg	75.00 (69.00, 82.00)	79.00 (72.00, 87.00)	83.00 (75.00, 90.00)	<0.001
WC, cm	82.00 (75.00, 88.00)	88.00 (81.00, 94.00)	93.00 (88.00, 99.00)	<0.001
BMI, kg/m^2^	23.24 (21.30, 25.06)	25.14 (22.95, 27.44)	26.84 (24.86, 29.07)	<0.001
Hb, g/L	144.00 (134.00, 156.00)	150.00 (138.00, 159.00)	153.00 (143.00, 161.00)	<0.001
FPG, mmol/L	5.64 (5.56, 5.77)	5.83 (5.66, 6.08)	6.35 (6.02, 6.63)	<0.001
TC, mmol/L	4.86 (4.29, 5.49)	5.05 (4.46, 5.70)	5.09 (4.48, 5.74)	<0.001
TG, mmol/L	1.26 (0.92, 1.72)	1.58 (1.13, 2.20)	1.91 (1.40, 2.63)	<0.001
HDL-C, mmol/L	1.36 (1.16, 1.59)	1.25 (1.06, 1.47)	1.15 (1.00, 1.33)	<0.001
LDL-C, mmol/L	2.67 (2.20, 3.19)	2.83 (2.36, 3.35)	2.82 (2.33, 3.32)	<0.001
CMI	0.44 (0.29, 0.67)	0.65 (0.41, 1.03)	0.90 (0.62, 1.36)	<0.001
AIP	−0.03 (−0.21, 0.14)	0.10 (−0.09, 0.28)	0.22 (0.05, 0.39)	<0.001
LAP	24.90 (15.39, 37.60)	39.33 (24.00, 61.44)	56.16 (38.50, 82.96)	<0.001
WWI	9.98 (9.58, 10.40)	10.29 (9.88, 10.71)	10.53 (10.17, 10.90)	<0.001
VAI	1.42 (0.97, 2.10)	1.87 (1.22, 2.84)	2.41 (1.63, 3.61)	<0.001
TyG	8.65 (8.33, 8.96)	8.91 (8.57, 9.25)	9.16 (8.85, 9.49)	<0.001
TyG-BMI	201.39 (181.03, 220.86)	224.03 (200.45, 249.04)	245.49 (224.89, 270.43)	<0.001
TyG-WC	706.57 (639.04, 770.62)	785.04 (708.67, 858.59)	852.85 (789.07, 919.27)	<0.001
WHtR	0.48 (0.45, 0.51)	0.52 (0.48, 0.55)	0.54 (0.52, 0.58)	<0.001
TyG-WHtR	4.20 (3.83, 4.52)	4.62 (4.21, 5.02)	5.01 (4.66, 5.36)	<0.001
BRI	3.05 (2.50–3.59)	3.68 (3.03–4.39)	4.23 (3.68–4.96)	<0.001
Sex, *n* (%)				<0.001
Male	6,610 (48.59)	12,170 (64.75)	2,334 (74.69)	
Female	6,995 (51.41)	6,625 (35.25)	791 (25.31)	
Smoking, *n* (%)				<0.001
No	13,088 (96.20)	18,032 (95.94)	2,919 (93.41)	
Yes	517 (3.80)	763 (4.06)	206 (6.59)	
Drinking, *n* (%)				<0.001
No	12,891 (94.75)	18,022 (95.89)	2,995 (95.84)	
Yes	714 (5.25)	773 (4.11)	130 (4.16)	
Medication use, *n* (%)				<0.001
No	12,946 (95.16)	16,386 (87.18)	2,400 (76.80)	
Yes	659 (4.84)	2,409 (12.82)	725 (23.20)	
Hypertension, *n* (%)				<0.001
No	11,700 (86.00)	13,207 (70.27)	1,763 (56.42)	
Yes	1,905 (14.00)	5,588 (29.73)	1,362 (43.58)	
Dyslipidemia, *n* (%)				<0.001
No	10,486 (77.07)	11,589 (61.66)	1,490 (47.68)	
Yes	3,119 (22.93)	7,206 (38.34)	1,635 (52.32)	

SBP, systolic blood pressure; DBP, diastolic blood pressure; WC, waist circumference; Hb, hemoglobin; FPG, fasting plasma glucose; TC, total cholesterol; TG, triglyceride; HDL-C, high-density lipoprotein cholesterol; LDL-C, low-density lipoprotein cholesterol; BMI, body mass index; CMI, cardiometabolic index; AIP, atherogenic index of plasma; LAP, lipid accumulation index; WWI, weight-adjusted-waist index; VAI, visceral adiposity index; BRI, body roundness index; WHtR, waist circumference/height; TyG, triglyceride-glucose index; TyG-BMI, triglycerideglucose × body mass index; TyG-WC, triglyceride-glucose × waist circumference; TyG-WHtR, triglyceride-glucose × waist circumference/height.

### 3.2 Association between 12 cardiometabolic indices and the progression to diabetes

**Figure 1:**
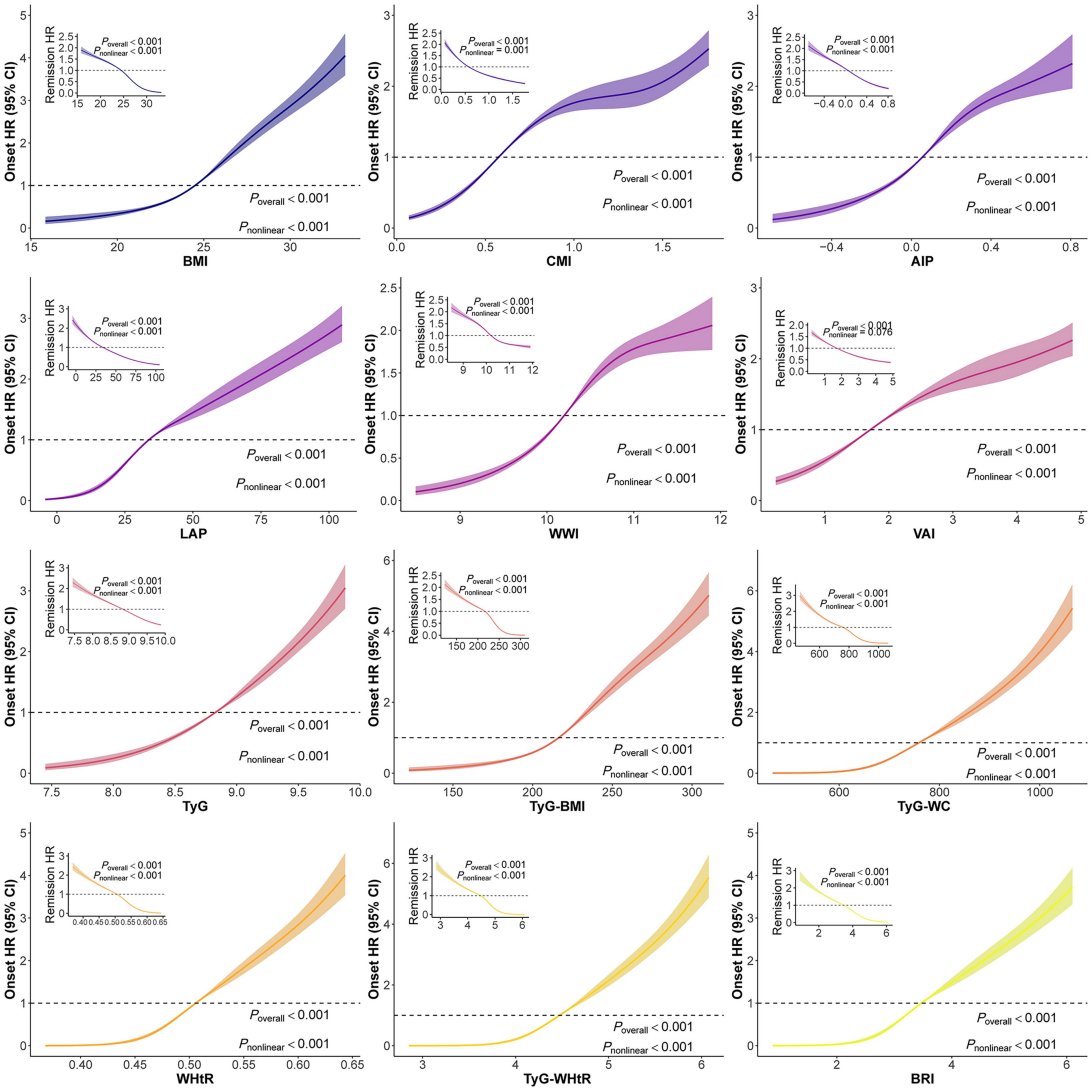
Restricted cubic spline analysis of 12 cardiometabolic indices with the progression to diabetes and the reversion to normoglycemia. BMI, body mass index; CMI, cardiometabolic index; AIP, atherogenic index of plasma; LAP, lipid accumulation index; WWI, weight-adjusted-waist index; VAI, visceral adiposity index; BRI, body roundness index; WHtR, waist circumference/height; TyG, triglyceride-glucose index; TyG-BMI, triglyceride glucose-body mass index; TyG-WC, triglyceride glucose-waist circumference; TyG-WHtR, triglyceride glucose-waist height ratio. The upper left curve represents the dose–response curve between 12 cardiometabolic indices and the reversion to normoglycemia. Bold lines represent HRs, while shaded areas indicate 95% CI.

The proportional hazards assumption was assessed using Schoenfeld residuals, and the overall tests indicated no evidence of violation for the Cox models used in this study (overall *p* > 0.05; Supplementary Table 3). RCS analyses revealed that all indices except VAI showed nonlinear associations with progression to diabetes and reversion to normoglycemia (overall *p* < 0.001; nonlinear *p* < 0.001; Figure. 1). Multicollinearity diagnostics yielded variance inflation factors (VIFs) <4 for all covariates (Supplementary Table 4). After adjustment for age, sex, dyslipidemia, hypertension, medication use, drinking, and smoking, every cardiometabolic index was significantly associated with progression to diabetes (*p* < 0.001). Adjusted HRs (Model 3) for progression were: BMI 1.79 (95% CI 1.72–1.86), CMI 1.66 (95% CI 1.59–1.74), AIP 1.63 (95% CI 1.55–1.72), LAP 1.75 (95% CI 1.68–1.82), WWI 1.44 (95% CI 1.39–1.50), VAI 1.53 (95% CI 1.46–1.60), WHtR 1.85 (95% CI 1.78–1.93), TyG 1.88 (95% CI 1.78–1.98), TyG-BMI 2.02 (95% CI 1.94–2.10), TyG-WC 2.21 (95% CI 2.11–2.31), TyG-WHtR 2.16 (95% CI 2.07–2.25), and BRI 1.79 (95% CI 1.72–1.85; Table 2).

**Table 2:** Association between 12 cardiometabolic indices and the progression to diabetes.

Variable	Model 1 HR(95%CI)	*p*-value	Model 2 HR(95%CI)	*p*-value	Model 3 HR(95%CI)	*p*-value
BMI	1.93 (1.86, 2.00)	<0.001	1.90 (1.83,1.97)	<0.001	1.79 (1.72,1.86)	<0.001
CMI	1.60 (1.56,1.65)	<0.001	1.59 (1.54,1.64)	<0.001	1.66 (1.59,1.74)	<0.001
AIP	1.64 (1.58,1.70)	<0.001	1.64 (1.58,1.70)	<0.001	1.63 (1.55,1.72)	<0.001
LAP	1.75 (1.70,1.81)	<0.001	1.70 (1.65,1.76)	<0.001	1.75 (1.68,1.82)	<0.001
WWI	1.64 (1.58,1.70)	<0.001	1.51 (1.46,1.57)	<0.001	1.44 (1.39,1.50)	<0.001
VAI	1.51 (1.46,1.55)	<0.001	1.52 (1.47,1.56)	<0.001	1.53 (1.46,1.60)	<0.001
WHtR	2.09 (2.02,2.17)	<0.001	1.96 (1.89,2.04)	<0.001	1.85 (1.78,1.93)	<0.001
TyG	1.84 (1.77,1.91)	<0.001	1.79 (1.72,1.86)	<0.001	1.88 (1.78,1.98)	<0.001
TyG-BMI	2.11 (2.03,2.18)	<0.001	2.11 (2.03,2.19)	<0.001	2.02 (1.94,2.10)	<0.001
TyG-WC	2.22 (2.14,2.30)	<0.001	2.26 (2.17,2.35)	<0.001	2.21 (2.11,2.31)	<0.001
TyG-WHtR	2.32 (2.24,2.41)	<0.001	2.20 (2.12,2.28)	<0.001	2.16 (2.07,2.25)	<0.001
BRI	2.01 (1.94,2.08)	<0.001	1.89 (1.82,1.96)	<0.001	1.79 (1.72,1.85)	<0.001

Model 1: Unadjusted. Model 2: Adjusted for age and sex. Model 3: Adjusted for age, sex, dyslipidemia, hypertension, medication use, drinking and smoking. HR, Hazard ratio; CI, Confidence interval; BMI, body mass index; CMI, cardiometabolic index; AIP, atherogenic index of plasma; LAP, lipid accumulation index; WWI, weight-adjusted-waist index; VAI, visceral adiposity index; BRI, body roundness index; WHtR, waist circumference/height; TyG, triglyceride-glucose index; TyG-BMI, triglyceride glucose-body mass index; TyG-WC, triglyceride glucose-waist circumference; TyG-WHtR, triglyceride glucose-waist height ratio.

### 3.3 Association between 12 cardiometabolic indices and the reversion to normoglycemia

After adjusting for variables such as age, sex, dyslipidemia, hypertension, medication use, drinking and smoking, every cardiometabolic index showed a significant association with reversion to normoglycemia (*p* < 0.001). Specifically, the HRs of the 12 indices for reversion to normoglycemia were as follows: BMI (HR = 0.68, 95% CI: 0.66–0.69), CMI (HR = 0.62, 95% CI: 0.61–0.64), AIP (HR = 0.75, 95% CI: 0.74–0.77), LAP (HR = 0.56, 95% CI: 0.55–0.58), WWI (HR = 0.84, 95% CI: 0.82–0.85), VAI (HR = 0.71, 95% CI: 0.69–0.73), WHtR (HR = 0.67, 95% CI: 0.65–0.68), TyG (HR = 0.74, 95% CI: 0.73–0.76), TyG-BMI (HR = 0.62, 95% CI: 0.61–0.63), TyG-WC (HR = 0.59, 95% CI: 0.57–0.60), TyG-WHtR (HR = 0.60, 95% CI: 0.59–0.62), and BRI (HR = 0.64, 95% CI: 0.63–0.66; Table 3).

**Table 3:** Association between 12 cardiometabolic indices and the reversion to normoglycemia.

Variable	Model 1 HR(95%CI)	*p*-value	Model 2 HR(95%CI)	*p*-value	Model 3 HR(95%CI)	*p*-value
BMI	0.59 (0.58, 0.60)	<0.001	0.65 (0.64, 0.66)	<0.001	0.68 (0.66, 0.69)	<0.001
CMI	0.58 (0.56, 0.59)	<0.001	0.63 (0.62, 0.65)	<0.001	0.62 (0.61, 0.64)	<0.001
AIP	0.67 (0.66, 0.69)	<0.001	0.73 (0.71, 0.74)	<0.001	0.75 (0.74, 0.77)	<0.001
LAP	0.50 (0.49, 0.51)	<0.001	0.57 (0.55, 0.58)	<0.001	0.56 (0.55, 0.58)	<0.001
WWI	0.70 (0.68, 0.71)	<0.001	0.81 (0.79, 0.82)	<0.001	0.84 (0.82, 0.85)	<0.001
VAI	0.67 (0.66, 0.69)	<0.001	0.70 (0.68, 0.71)	<0.001	0.71 (0.69, 0.73)	<0.001
WHtR	0.56 (0.55, 0.57)	<0.001	0.64 (0.62, 0.65)	<0.001	0.67 (0.65, 0.68)	<0.001
TyG	0.66 (0.64, 0.67)	<0.001	0.72 (0.71, 0.73)	<0.001	0.74 (0.73, 0.76)	<0.001
TyG-BMI	0.54 (0.53, 0.55)	<0.001	0.60 (0.59, 0.61)	<0.001	0.62 (0.61, 0.63)	<0.001
TyG-WC	0.53 (0.52, 0.54)	<0.001	0.57 (0.56, 0.58)	<0.001	0.59 (0.57, 0.60)	<0.001
TyG-WHtR	0.52 (0.51, 0.53)	<0.001	0.59 (0.57, 0.60)	<0.001	0.60 (0.59, 0.62)	<0.001
BRI	0.54 (0.53, 0.55)	<0.001	0.61 (0.60, 0.63)	<0.001	0.64 (0.63, 0.66)	<0.001

Model 1: Unadjusted. Model 2: Adjusted for age and sex. Model 3: Adjusted for age, sex, dyslipidemia, hypertension, medication use, drinking and smoking. HR, Hazard ratio; CI, Confidence interval; BMI, body mass index; CMI, cardiometabolic index; AIP, atherogenic index of plasma; LAP, lipid accumulation index; WWI, weight-adjusted-waist index; VAI, visceral adiposity index; BRI, body roundness index; WHtR, waist circumference/height; TyG, triglyceride-glucose index; TyG-BMI, triglyceride glucose-body mass index; TyG-WC, triglyceride glucose-waist circumference; TyG-WHtR, triglyceride glucose-waist height ratio.

### 3.4 Predictive performance comparison

**Figure 2:**
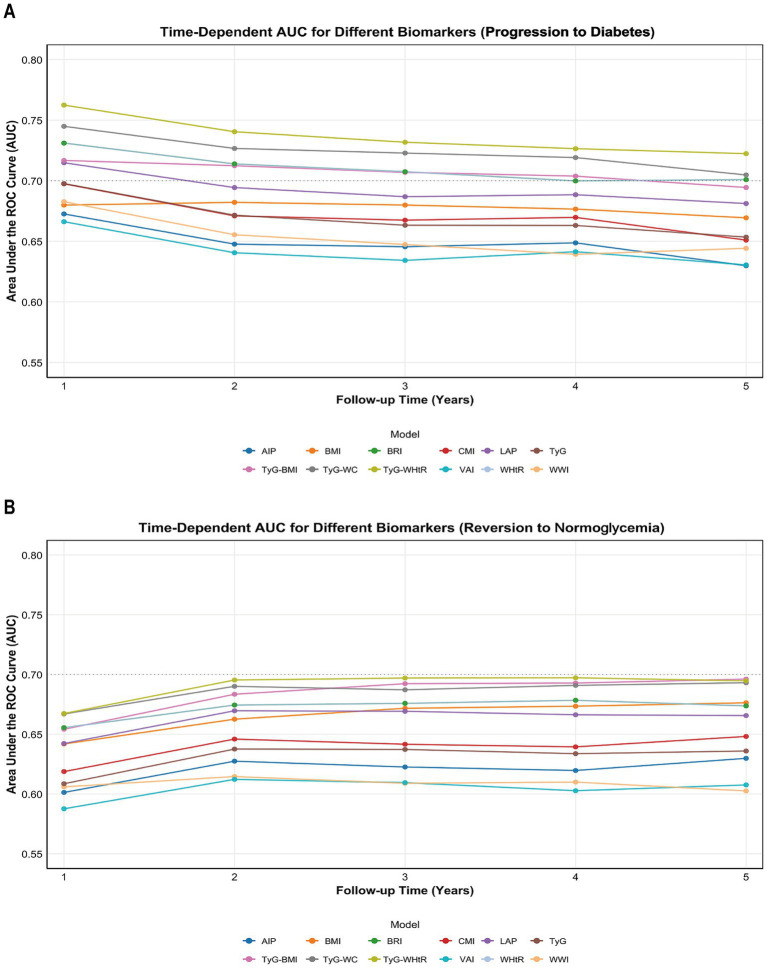
Time-dependent predictive performance of 12 cardiometabolic indices for predicting the progression to diabetes **(A)** and the reversion to normoglycemia **(B)**. BMI, body mass index; CMI, cardiometabolic index; AIP, atherogenic index of plasma; LAP, lipid accumulation index; WWI, weight-adjusted-waist index; VAI, visceral adiposity index; BRI, body roundness index; WHtR, waist circumference/height; TyG, triglyceride-glucose index; TyG-BMI, triglyceride glucose-body mass index; TyG-WC, triglyceride glucose-waist circumference; TyG-WHtR, triglyceride glucose-waist height ratio.

Time-dependent ROC curves showed that TyG-WHtR had the highest AUC for discriminating progression to diabetes and reversion to normoglycemia (Figure 2). The C-index, NRI, and IDI further supported the relatively better discriminatory performance of TyG-WHtR. For progression to diabetes, the C-index was 0.747 (95% CI 0.739–0.756), the NRI was 0.315 (95% CI 0.119–0.471), and the IDI was 0.039 (95% CI 0.012–0.068). For reversion to normoglycemia, the C-index was 0.673 (95% CI 0.668–0.677), the NRI was 0.199 (95% CI 0.099–0.252), and the IDI was 0.056 (95% CI 0.028–0.077). TyG-WC and TyG-BMI ranked next (C-index: 0.734 and 0.720 for progression, and 0.668 and 0.661 for reversion, respectively). Common anthropometric indices, including BRI, WHtR, and BMI, showed moderate discriminatory performance. Notably, in the progression model, the TyG index had a comparatively modest C-index (0.680) but the highest NRI (0.329) and IDI (0.055) (Supplementary Tables 5, 6 and Supplementary Figures 4, 5).

### 3.5 Mediating effects of blood biomarkers

**Figure 3:**
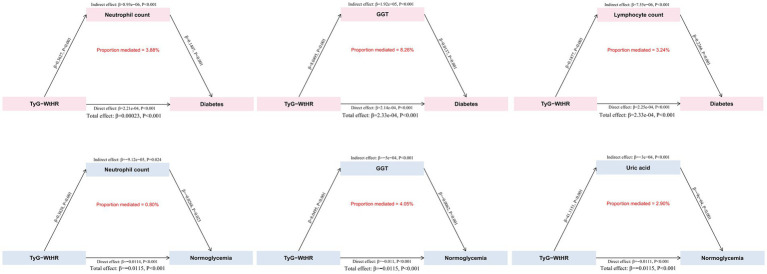
Mediation analysis of TyG-WHtR with progression to diabetes and reversion to normoglycemia. The upper panel shows mediation models with progression to diabetes as the outcome; the lower panel shows mediation models with reversion to normoglycemia as the outcome. GGT, gamma-glutamyl transpeptidase; TyG-WHtR, triglyceride glucose-waist height ratio.

Given that the TyG-WHtR index demonstrated relatively high predictive value among the 12 cardiometabolic indices for predicting progression to diabetes and reversion to normoglycemia, it was selected as the exposure variable for the mediational analysis to investigate potential indirect pathways involving specific biomarkers. As shown in Supplementary Table 7, the TyG-WHtR index exhibited significant correlations with all selected biomarkers except WBC (*p* < 0.001). The associations between these biomarkers and the progression to diabetes and reversion to normoglycemia are presented in Supplementary Table 8. Among the six selected biomarkers, four were significantly associated with both progression to diabetes and reversion to normoglycemia (*p* < 0.001): neutrophil count, lymphocyte count, GGT, and uric acid. After excluding pathways that did not reach statistical significance (*p* > 0.05), the final analysis included three biomarkers demonstrating statistically significant but modest indirect associations (all *p* < 0.05; Supplementary Table 9). Specifically, regarding the association between the TyG-WHtR index and progression to diabetes, the proportion accounted for by lymphocyte count was 3.24% (95% CI 2.25–4.31%), by neutrophil count was 3.88% (95% CI 2.86–4.95%), and by GGT was 8.26% (95% CI 6.61–9.77%). Similarly, for the association between the TyG-WHtR index and reversion to normoglycemia, the proportions explained by neutrophil count, uric acid, and GGT were 0.80% (95% CI 0.11–1.46%), 2.90% (95% CI 1.78–4.03%), and 4.05% (95% CI 2.85–5.26%), respectively (Figure 3; Supplementary Table 9).

### 3.6 Subgroup and sensitivity analyses

TyG-WHtR was selected for subgroup analyses because it showed relatively better discriminatory performance. Positive associations with progression to diabetes and negative associations with reversion to normoglycemia were generally consistent across subgroups. Exploratory analyses suggested somewhat stronger associations with progression in younger individuals, those with baseline FPG < 6.1 mmol/L, those without baseline medication use, and those without obesity, dyslipidemia, or hypertension (Supplementary Figure 6A). For reversion to normoglycemia, exploratory analyses suggested somewhat stronger associations in males, those with baseline FPG 6.1–6.9 mmol/L, those with baseline medication use, and those with obesity, dyslipidemia, or hypertension (Supplementary Figure 6B). These subgroup findings should be interpreted cautiously because of their exploratory nature, the possibility of multiple testing, and reduced precision after stratification. In sensitivity analyses, results were similar after redefining prediabetes using the WHO criteria (*n* = 28,253), after excluding participants lost to follow-up or with follow-up duration <2 years (*n* = 31,732), and after excluding participants with any baseline medication use (*n* = 17,641; Supplementary Tables 10–15).

## 4 Discussion

This prospective cohort study of 35,525 participants assessed 12 cardiometabolic indices and identified nonlinear associations with both progression from prediabetes to diabetes and reversion to normoglycemia. All indices were significantly associated with increased risk of progression and decreased likelihood of reversion after adjusting for confounders. TyG-WHtR showed relatively better discriminatory performance for both outcomes. Mediation analyses suggested that inflammation- and oxidative stress-related biomarkers may represent potential indirect pathways linking TyG-WHtR with glycemic transitions. Subgroup analyses showed generally consistent associations across strata, although those findings should be interpreted cautiously.

Previous studies have primarily focused on prediabetes progression to diabetes (26, 27). However, prediabetes reversal is equally important, as it supports earlier identification of at-risk individuals and delays the onset of diabetes and its complications. A recent retrospective cohort study, aligned with ADA standards, followed two prediabetes cohorts from Chinese medical centers over 5 years, reporting progression rates to diabetes of 41.6 and 35.2% in the initial and external cohorts, respectively, with annual rates of 8.34 and 7.04% (28). Additionally, a 2022 meta-analysis of 35 randomized controlled trials (RCTs) found that 31% of participants reverted from prediabetes to normoglycemia within 1.6 years (29). Unlike prior studies, our study places equal emphasis on prediabetes progression and reversal and examines their associations with cardiometabolic indices to identify the indicator with relatively better discriminatory performance. The annual diabetes progression rate observed here was broadly consistent with previous research. Given the large sample size and the focus on participants with prediabetes at enrollment, the incidence of reversion to normoglycemia in this study was slightly higher than that reported in the meta-analysis but remained within a plausible range.

While many studies have explored the link between cardiometabolic indices and diabetes, most have been limited by small sample sizes or have focus on individual biomarkers. To date, few studies have systematically compared these cardiometabolic indices with respect to both progression from prediabetes to diabetes and reversion to normoglycemia. Although most research has investigated CMI as a metabolic marker combining blood lipid and obesity parameters to predict various diseases, including cardiovascular disease, it was originally developed to distinguish diabetes (12, 30). AIP is a novel indicator for assessing blood lipid levels. A cohort study demonstrated the clinical utility of AIP in long-term risk assessment using explainable machine learning methods, underscoring its value as a reliable predictor in longitudinal observations (31). AIP has also been studied in relation to insulin resistance and type 2 diabetes (32), and several other indices, such as VAI, BRI, and LAP, have also been proposed as useful markers of metabolic risk (33, 34). Recent studies have examined the TyG index and its composite derivatives in relation to diabetes risk. For example, a retrospective study involving 6,072 Korean adults with fasting blood glucose abnormalities demonstrated that the TyG index predicted type 2 diabetes more effectively than oral glucose tolerance test-derived insulin sensitivity and secretion markers (35). Other studies have reported that TyG-related composite indicators may offer additional discriminatory information (36, 37), and a Chinese study in older adults suggested that TyG-WHtR showed relatively better predictive performance than several related parameters for incident diabetes (38). These observations are broadly consistent with our findings.

Although this study supports associations between cardiometabolic markers and glycemic transitions, the biological mechanisms underlying these outcomes remain incompletely understood. Our mediation analyses suggest that inflammation- and oxidative stress-related biomarkers may be involved in potential indirect pathways. GGT is an indicator of hepatic lipid accumulation, and fatty liver deposition may increase inflammatory cytokines and oxidative stress, which are closely associated with insulin resistance (IR), a key factor in diabetes pathogenesis (39). Experimental studies have also shown that GGT plays a role in intracellular antioxidant defense through its mediation of extracellular glutathione transport into cells (40). Thus, elevated GGT may reflect oxidative stress relevant to the development of diabetes (40, 41). Similarly, elevated triglycerides and glucose can impair beta-cell function and intensify inflammatory responses. Hyperglycemia may increase reactive oxygen species production and trigger endoplasmic reticulum stress and mitochondrial dysfunction, while elevated triglycerides and free fatty acids may exacerbate insulin resistance and beta-cell dysfunction (42–45). Obesity, particularly visceral adiposity, may further contribute to chronic inflammation and insulin resistance through adipocytokine imbalance (20, 46–48). Taken together, these mechanisms may help explain why TyG-WHtR, which integrates triglycerides, glycemia, and central adiposity, showed relatively better discriminatory performance in this cohort. However, because the exposure and candidate mediators were measured at the same time point in an observational study, these mediation findings should be interpreted as exploratory and should not be considered causal.

Furthermore, we further explored whether the associations differed across clinically relevant subgroups. Previous studies have reported inconsistent findings regarding the influence of age and sex on prediabetes transitions (38, 49–51). Moreover, modifiable factors such as obesity, dyslipidemia, and hypertension also significantly influence the outcome of prediabetes (51). We also performed subgroup analyses by baseline FPG and medication use because baseline glycemic status and treatment may influence both cardiometabolic indices and the likelihood of subsequent progression or reversion (52, 53). In our study, some heterogeneity was observed across age, baseline FPG, BMI, dyslipidemia, hypertension, and medication-use strata. However, these subgroup analyses were exploratory and may reflect true heterogeneity, residual confounding, reduced statistical precision after stratification, or multiple testing. Therefore, they should not be overinterpreted.

This study has several strengths, including the systematic comparison of 12 cardiometabolic indices across both prediabetes progression and reversion. However, several limitations should be acknowledged. First, this was a single-center study, and the participants were primarily from northern China, which may limit the generalizability of the findings; further validation is needed in multicenter cohorts, in other geographic regions, and across diverse ethnic populations. Second, as an observational study, residual confounding may persist because important variables such as diet, physical activity, socioeconomic status, and family history of diabetes were unavailable. Accordingly, the present findings should be interpreted as observational associations rather than causal effects. In addition, medication use in this cohort was broadly defined and could not be categorized more precisely because the source records were heterogeneous and incomplete, which may limit the assessment of drug-specific effects. Third, this study focused on comparing the associations and relative discriminatory performance of different cardiometabolic indices rather than developing a full clinical prediction model; therefore, calibration was not assessed, and an optimal cut-off value for TyG-WHtR was not determined. Future work could integrate novel cardiometabolic biomarkers with conventional risk factors and machine learning approaches to derive clinically useful thresholds. Fourth, because HbA1c was not available for all participants during routine health examinations, reversion to normoglycemia was defined using FPG alone, whereas diabetes and prediabetes were defined using both FPG and HbA1c. This inconsistency may have introduced some misclassification and could have led to overestimation of reversion to normoglycemia. Fifth, only baseline cardiometabolic parameters were analyzed; dynamic changes over time were not modeled, and therefore the findings should not be interpreted as definitive long-term predictive performance. Finally, this study was based on periodic health examinations, so all outcomes and loss to follow-up were determined at scheduled visits rather than at exact occurrence times. The observed event time therefore reflected the first detection date rather than the exact biological onset time, giving the data an interval-censored structure. Under these conditions, standard competing-risk models such as the Fine-Gray model may be difficult to apply reliably, especially because accurate mortality timing and cause-of-death data were unavailable in the database. For this reason, the present results should be interpreted as associations observed within a follow-up framework rather than as strict survival-time inference.

## 5 Conclusion

Cardiometabolic indices were positively associated with progression from prediabetes to diabetes and negatively associated with reversion to normoglycemia. Inflammation and oxidative stress may represent potential pathways underlying these associations. Among the evaluated indicators, TyG-WHtR showed relatively stronger and more consistent associations, as well as relatively better discriminatory performance in this cohort, suggesting potential utility as a noninvasive marker for risk stratification in prediabetes.

## Data Availability

The original contributions presented in the study are included in the article/Supplementary material, further inquiries can be directed to the corresponding author.

## References

[1] Echouffo-Tcheugui JB Selvin E. Prediabetes and what it means: the epidemiological evidence. Annu Rev Public Health. (2021) 42:59–77. doi: 10.1146/annurev-publhealth-090419-102644, 33355476 PMC8026645

[2] Tabák AG Herder C Rathmann W Brunner EJ Kivimäki M. Prediabetes: a high-risk state for diabetes development. Lancet. (2012) 379:2279–90. doi: 10.1016/s0140-6736(12)60283-9, 22683128 PMC3891203

[3] Wang L Peng W Zhao Z Zhang M Shi Z Song Z . Prevalence and treatment of diabetes in China, 2013-2018. JAMA. (2021) 326:2498–506. doi: 10.1001/jama.2021.22208, 34962526 PMC8715349

[4] Li Y Teng D Shi X Qin G Qin Y Quan H . Prevalence of diabetes recorded in mainland China using 2018 diagnostic criteria from the American Diabetes Association: national cross sectional study. BMJ. (2020) 369:m997. doi: 10.1136/bmj.m997, 32345662 PMC7186854

[5] Richter B Hemmingsen B Metzendorf MI Takwoingi Y. Development of type 2 diabetes mellitus in people with intermediate hyperglycaemia. Cochrane Database Syst Rev. (2018) 2018:CD012661. doi: 10.1002/14651858.CD012661.pub2, 30371961 PMC6516891

[6] Hu B Li J Su H Wu J Gao Y Yang H . Global assessment of atherosclerotic cardiovascular disease quality of care index in adults aged ≥60 years. Nutr Metab Cardiovasc Dis. (2026) 36:104422. doi: 10.1016/j.numecd.2025.104422, 41241629

[7] Wallace AS Rooney MR Fang M Echouffo-Tcheugui JB Grams M Selvin E. Natural history of prediabetes and long-term risk of clinical outcomes in middle-aged adults: the atherosclerosis risk in communities (ARIC) study. Diabetes Care. (2023) 46:e67–8. doi: 10.2337/dc22-1321, 36525570 PMC9887606

[8] Bailey CJ. Prediabetes: never too early to intervene. Lancet Diabetes Endocrinol. (2023) 11:529–30. doi: 10.1016/s2213-8587(23)00152-3, 37414070

[9] Chakraborty S Verma A Garg R Singh J Verma H. Cardiometabolic risk factors associated with type 2 diabetes mellitus: a mechanistic insight. Clin Med Insights Endocrinol Diabetes. (2023) 16:11795514231220780. doi: 10.1177/11795514231220780, 38148756 PMC10750528

[10] Xu B Wu Q Yin G Lu L la R Zhang Y . Associations of cardiometabolic index with diabetic statuses and insulin resistance: the mediating role of inflammation-related indicators. BMC Public Health. (2024) 24:2736. doi: 10.1186/s12889-024-20048-0, 39379887 PMC11460066

[11] He HM Xie YY Chen Q Li YK Li XX Mu YK . The additive effect of the triglyceride-glucose index and estimated glucose disposal rate on long-term mortality among individuals with and without diabetes: a population-based study. Cardiovasc Diabetol. (2024) 23:307. doi: 10.1186/s12933-024-02396-8, 39175051 PMC11342524

[12] Wakabayashi I Daimon T. The “cardiometabolic index” as a new marker determined by adiposity and blood lipids for discrimination of diabetes mellitus. Clin Chim Acta. (2015) 438:274–8. doi: 10.1016/j.cca.2014.08.042 25199852

[13] Dobiásová M. Atherogenic index of plasma [log(triglycerides/HDL-cholesterol)]: theoretical and practical implications. Clin Chem. (2004) 50:1113–5. doi: 10.1373/clinchem.2004.033175, 15229146

[14] Huang Q Liu Z Wei M Huang Q Feng J Liu Z . The atherogenic index of plasma and carotid atherosclerosis in a community population: a population-based cohort study in China. Cardiovasc Diabetol. (2023) 22:125. doi: 10.1186/s12933-023-01839-y, 37244995 PMC10225098

[15] Cai X Hu J Wen W Wang J Wang M Liu S . Associations of the cardiometabolic index with the risk of cardiovascular disease in patients with hypertension and obstructive sleep Apnea: results of a longitudinal cohort study. Oxidative Med Cell Longev. (2022) 2022:4914791. doi: 10.1155/2022/4914791, 35783191 PMC9246614

[16] Cui C Qi Y Song J Shang X Han T Han N . Comparison of triglyceride glucose index and modified triglyceride glucose indices in prediction of cardiovascular diseases in middle aged and older Chinese adults. Cardiovasc Diabetol. (2024) 23:185. doi: 10.1186/s12933-024-02278-z, 38812015 PMC11138075

[17] Song Z Miao X Liu S Hu M Xie X Sun Y . Associations between cardiometabolic indices and the onset of metabolic dysfunction-associated steatotic liver disease as well as its progression to liver fibrosis: a cohort study. Cardiovasc Diabetol. (2025) 24:154. doi: 10.1186/s12933-025-02716-6, 40181314 PMC11969729

[18] Wang Q Miao X Hu M Xu F Tang G He Y . Nonlinear relationship between serum 25-hydroxyvitamin D and lipid profile in Chinese adults. Front Nutr. (2024) 11:1388017. doi: 10.3389/fnut.2024.1388017, 38933885 PMC11199867

[19] Herder C Karakas M Koenig W. Biomarkers for the prediction of type 2 diabetes and cardiovascular disease. Clin Pharmacol Ther. (2011) 90:52–66. doi: 10.1038/clpt.2011.93 21654741

[20] Ahmed B Sultana R Greene MW. Adipose tissue and insulin resistance in obese. Biomed Pharmacother. (2021) 137:111315. doi: 10.1016/j.biopha.2021.111315 33561645

[21] Han M Huang K Shen C Hu H Liu F Li J . Discordant high remnant cholesterol with LDL-C increases the risk of stroke: a Chinese prospective cohort study. Stroke. (2024) 55:2066–74. doi: 10.1161/strokeaha.124.046811, 39038095

[22] Al-Makki A DiPette D Whelton PK Murad MH Mustafa RA Acharya S . Hypertension pharmacological treatment in adults: a World Health Organization guideline executive summary. Hypertension. (2022) 79:293–301. doi: 10.1161/HYPERTENSIONAHA.121.18192, 34775787 PMC8654104

[23] Suh SH Kim SW. Dyslipidemia in patients with chronic kidney disease: an updated overview. Diabetes Metab J. (2023) 47:612–29. doi: 10.4093/dmj.2023.0067, 37482655 PMC10555535

[24] Tian Z Yang L Li Y Huang Y Yang J Xue F. Associations of different insulin resistance-related indices with the incidence and progression trajectory of cardiometabolic multimorbidity: a prospective cohort study from UK biobank. Cardiovasc Diabetol. (2025) 24:257. doi: 10.1186/s12933-025-02819-0, 40533754 PMC12175334

[25] Organization WH. Definition and Diagnosis of Diabetes Mellitus and Intermediate Hyperglycaemia: Report of a WHO/IDF Consultation. Geneva: World Health Organization (2006).

[26] Yokota N Miyakoshi T Sato Y Nakasone Y Yamashita K Imai T . Predictive models for conversion of prediabetes to diabetes. J Diabetes Complicat. (2017) 31:1266–71. doi: 10.1016/j.jdiacomp.2017.01.005, 28173983

[27] Chan JC Zhang Y Ning G. Diabetes in China: a societal solution for a personal challenge. Lancet Diabetes Endocrinol. (2014) 2:969–79. doi: 10.1016/s2213-8587(14)70144-5, 25218728

[28] Zhang Y Zhang H Wang D Li N Lv H Zhang G. Development of a 5-year risk prediction model for transition from prediabetes to diabetes using machine learning: retrospective cohort study. J Med Internet Res. (2025) 27:e73190. doi: 10.2196/73190, 40344663 PMC12102623

[29] Galaviz KI Weber MB Suvada KB Gujral UP Wei J Merchant R . Interventions for reversing prediabetes: a systematic review and Meta-analysis. Am J Prev Med. (2022) 62:614–25. doi: 10.1016/j.amepre.2021.10.020, 35151523 PMC10420389

[30] Li J Wei X. Baseline and changes in cardiometabolic index and incident cardiovascular disease in two prospective cohorts. Am J Prev Cardiol. (2025) 23:101046. doi: 10.1016/j.ajpc.2025.101046, 40612280 PMC12226060

[31] Hu B Wang Y Li Z Sun H Ren Z Hu H . The atherogenic index of plasma predicts long-term outcomes in patients with severe coronary artery calcification undergoing rotational atherectomy: a machine learning-based cohort study. Cardiovasc Diabetol. (2025) 25:2. doi: 10.1186/s12933-025-03027-6, 41327344 PMC12777130

[32] Yin B Wu Z Xia Y Xiao S Chen L Li Y. Non-linear association of atherogenic index of plasma with insulin resistance and type 2 diabetes: a cross-sectional study. Cardiovasc Diabetol. (2023) 22:157. doi: 10.1186/s12933-023-01886-5, 37386500 PMC10311747

[33] Ji B Qu H Wang H Wei H Deng H. Association between the visceral adiposity index and homeostatic model assessment of insulin resistance in participants with normal waist circumference. Angiology. (2017) 68:716–21. doi: 10.1177/0003319716682120, 28743220

[34] Zhao Q Zhang K Li Y Zhen Q Shi J Yu Y . Capacity of a body shape index and body roundness index to identify diabetes mellitus in Han Chinese people in Northeast China: a cross-sectional study. Diabet Med. (2018) 35:1580–7. doi: 10.1111/dme.13787, 30059165

[35] Lee MJ Bae JH Khang AR Yi D Yun MS Kang YH. Triglyceride-glucose index predicts type 2 diabetes mellitus more effectively than oral glucose tolerance test-derived insulin sensitivity and secretion markers. Diabetes Res Clin Pract. (2024) 210:111640. doi: 10.1016/j.diabres.2024.111640, 38548110

[36] Li X Li G Cheng T Liu J Song G Ma H. Association between triglyceride-glucose index and risk of incident diabetes: a secondary analysis based on a Chinese cohort study: TyG index and incident diabetes. Lipids Health Dis. (2020) 19:236. doi: 10.1186/s12944-020-01403-7, 33161902 PMC7649000

[37] Ke P Wu X Xu M Feng J Xu H Gan Y . Comparison of obesity indices and triglyceride glucose-related parameters to predict type 2 diabetes mellitus among normal-weight elderly in China. Eat Weight Disord. (2022) 27:1181–91. doi: 10.1007/s40519-021-01238-w 34195936

[38] Li X Sun M Yang Y Yao N Yan S Wang L . Predictive effect of triglyceride glucose-related parameters, obesity indices, and lipid ratios for diabetes in a Chinese population: a prospective cohort study. Front Endocrinol (Lausanne). (2022) 13:862919. doi: 10.3389/fendo.2022.862919, 35432185 PMC9007200

[39] Liu D Li J Lu W Wang Y Zhou X Huang D . Gamma-glutamyl transpeptidase to cholinesterase and platelet ratio in predicting significant liver fibrosis and cirrhosis of chronic hepatitis B. Clin Microbiol Infect. (2019) 25:514.e1–8. doi: 10.1016/j.cmi.2018.06.002, 29906588

[40] Karp DR Shimooku K Lipsky PE. Expression of gamma-glutamyl transpeptidase protects Ramos B cells from oxidation-induced cell death. J Biol Chem. (2001) 276:3798–804. doi: 10.1074/jbc.M008484200, 11080500

[41] Lee DH Ha MH Kim JH Christiani DC Gross MD Steffes M . Gamma-glutamyltransferase and diabetes--a 4 year follow-up study. Diabetologia. (2003) 46:359–64. doi: 10.1007/s00125-003-1036-5 12687334

[42] Holendová B Šalovská B Benáková Š Plecitá-Hlavatá L. Beyond glucose: the crucial role of redox signaling in β-cell metabolic adaptation. Metabolism. (2024) 161:156027. doi: 10.1016/j.metabol.2024.156027, 39260557

[43] Lee JH Lee J. Endoplasmic reticulum (ER) stress and its role in pancreatic β-cell dysfunction and senescence in type 2 diabetes. Int J Mol Sci. (2022) 23:4843. doi: 10.3390/ijms23094843, 35563231 PMC9104816

[44] Søndergaard E Nielsen S. VLDL triglyceride accumulation in skeletal muscle and adipose tissue in type 2 diabetes. Curr Opin Lipidol. (2018) 29:42–7. doi: 10.1097/mol.0000000000000471, 29135689

[45] Guo H Ma C Wu X Pan C. Functional status of pancreatic α and β cells in type 2 diabetes mellitus patients with different plasma triglyceride levels: a retrospective analysis. Int J Endocrinol. (2021) 2021:1–6. doi: 10.1155/2021/9976067, 34457002 PMC8387189

[46] Jung I Koo DJ Lee WY. Insulin resistance, non-alcoholic fatty liver disease and type 2 diabetes mellitus: clinical and experimental perspective. Diabetes Metab J. (2024) 48:327–39. doi: 10.4093/dmj.2023.0350, 38310873 PMC11140401

[47] Han J Kim SH Suh YJ Lim HA Shin H Cho SG . Serum chemerin levels are associated with abdominal visceral fat in type 2 diabetes. J Korean Med Sci. (2016) 31:924–31. doi: 10.3346/jkms.2016.31.6.924, 27247502 PMC4853672

[48] Song J Li Y Zhu J Liang J Xue S Zhu Z. Non-linear associations of cardiometabolic index with insulin resistance, impaired fasting glucose, and type 2 diabetes among US adults: a cross-sectional study. Front Endocrinol (Lausanne). (2024) 15:1341828. doi: 10.3389/fendo.2024.1341828, 38410697 PMC10894973

[49] Giráldez-García C Cea-Soriano L Albaladejo R Franch-Nadal J Mata-Cases M Díez-Espino J . The heterogeneity of reversion to normoglycemia according to prediabetes type is not explained by lifestyle factors. Sci Rep. (2021) 11:9667. doi: 10.1038/s41598-021-87838-z, 33958606 PMC8102601

[50] Perreault L Pan Q Mather KJ Watson KE Hamman RF Kahn SE. Effect of regression from prediabetes to normal glucose regulation on long-term reduction in diabetes risk: results from the diabetes prevention program outcomes study. Lancet. (2012) 379:2243–51. doi: 10.1016/s0140-6736(12)60525-x, 22683134 PMC3555407

[51] Davoodian N Lotfaliany M Huxley RR Lee CMY Pasco JA Adams RJ . Prediabetes transitions to normoglycaemia or type 2 diabetes and associated risk factors in the obesity, diabetes and cardiovascular disease collaboration: an individual-level pooled analysis of 19 prospective cohort studies. Lancet Glob Health. (2025) 13:e1533–42. doi: 10.1016/s2214-109x(25)00237-2, 40845880

[52] Sujana C Seissler J Jordan J Rathmann W Koenig W Roden M . Associations of cardiac stress biomarkers with incident type 2 diabetes and changes in glucose metabolism: KORA F4/FF4 study. Cardiovasc Diabetol. (2020) 19:178. doi: 10.1186/s12933-020-01117-1, 33066780 PMC7566143

[53] Liu X Tan Z Huang Y Zhao H Liu M Yu P . Relationship between the triglyceride-glucose index and risk of cardiovascular diseases and mortality in the general population: a systematic review and meta-analysis. Cardiovasc Diabetol. (2022) 21:124. doi: 10.1186/s12933-022-01546-0, 35778731 PMC9250255

