# Supplementary material for: Predictive performance of cardiometabolic indices for prediabetes progression to diabetes and reversion to normoglycemia in adults: a cohort study
Source: Front Nutr. 2026 May 14;13:1784247. doi: 10.3389/fnut.2026.1784247 (PMC13215993; doi:10.3389/fnut.2026.1784247)

**Supplementary Materials**

**Supplemental Table 1** Calculation formulas of twelve cardiometabolic indices.

**Supplemental Table 2** Baseline characteristics of selected biomarkers (n=35,525).

**Supplemental Table 3** Schoenfeld Residuals Test for the proportional hazards Assumption.

**Supplemental Table 4** Ulticollinearity Test using Variance Inflation Factor.

**Supplemental Table 5** The Predictive Value of 5-year twelve cardiometabolic indices for the reversion to normoglycemia.

**Supplemental Table 6** The Predictive Value of 5-year twelve cardiometabolic indices for the progression to diabetes.

**Supplemental Table 7** Associations between TyG-WHtR indices and selected mediators.

**Supplemental Table 8** Association between selected mediators and risk of progression to diabetes and reversion to normoglycemia.

**Supplemental Table 9** Mediated role of selected biomarkers in association between TyG-WHtR indices and risk of progression to diabetes and reversion to normoglycemia.

**Supplemental Table 10** Association between twelve cardiometabolic indices and the progression to diabetes after excluding individuals with FPG <6.1 mmol/L according to WHO recommended criteria.

**Supplemental Table 11** Association between twelve cardiometabolic indices and the reversion to normoglycemia after excluding individuals with FPG <6.1 mmol/L according to WHO recommended criteria.

**Supplemental Table 12** Association between twelve cardiometabolic indices and the progression to diabetes after excluding individuals who were lost to follow-up or with follow-up duration <2 years.

**Supplemental Table 13** Association between twelve cardiometabolic indices and the reversion to normoglycemia after excluding individuals who were lost to follow-up or with follow-up duration <2 years.

**Supplemental Table 14** Association between twelve cardiometabolic indices and the progression to diabetes after excluding individuals with any self-reported current medication use at baseline.

**Supplemental Table 15** Association between twelve cardiometabolic indices and the reversion to normoglycemia after excluding individuals with any self-reported current medication use at baseline.

**Supplemental Figure 1.** Flow chart of study subjects.

**Supplemental Figure 2.** Summary of missing variables.

**Supplemental Figure 3.** The final constructed directed acyclic graph.

**Supplemental Figure 4.**The Predictive Value of 5-year twelve cardiometabolic indices for the progression to diabetes as well as reversion to normoglycemia.

**Supplemental Figure 5.** The Predictive Value of 5-year twelve cardiometabolic indices for the reversion to normoglycemia.

**Supplemental Figure 6.** Subgroup analyses of the association of TyG-WHtR with progression to diabetes (A) and reversion to normoglycemia (B).

**Supplemental Table 1** Calculation formulas of twelve cardiometabolic indices.

| **Composite indicator** | **Formula** |
| --- | --- |
| BMI | weight (kg) / height (m)² |
| CMI | TG (mmol/L)/HDL-C (mmol/L)×WC (cm)/height (cm) |
| AIP | log10[TG(mmol/L)/HDL-C (mmol/L)] |
| LAP | (WC(cm)-65)×TG(mmol/L)(Males);  (WC(cm)-58)×TG(mmol/L)(Females) |
| WWI | WC (cm)/√weight (kg) |
| VAI | (WC / (39.68 + 1.88×BMI))×(TG / 1.03)×[1.31 / HDL-C(mmol/L)](Males);  (WC / (36.58 + 1.89×BMI))×(TG / 0.81)×[1.52 / HDL-C(mmol/L)](Females) |
| WHtR | WC (cm)/height (cm) |
| BRI | $\text{364.2-365.5×}\sqrt{\text{1-(}\frac{\text{(WC(cm)/(2π))}^{\text{2}}}{\text{(0.5height(cm))}^{\text{2}}}\text{)}}$ |
| TyG | ln[TG (mg/dL)×FPG (mg/dL)/2 ] |
| TyG-BMI | TyG×BMI |
| TyG-WC | TyG×WC |
| TyG-WHtR | TyG×WHtR |

**Abbreviations:** TG, triglyceride; HDL-C, high-density lipoprotein cholesterol; WC, waist circumference; FPG, fasting plasma glucose; BMI, body mass index; CMI, cardiometabolic index; AIP, atherogenic index of plasma; LAP, lipid accumulation index; WWI, weight-adjusted-waist index; VAI, visceral adiposity index; BRI, body roundness index; WHtR, waist circumference/height; TyG, triglyceride-glucose index; TyG-BMI, triglyceride glucose-body mass index; TyG-WC, triglyceride glucose-waist circumference; TyG-WHtR, triglyceride glucose-waist height ratio.

**Supplemental Table 2** Baseline characteristics of selected biomarkers (n=35,525).

| **Mediators** | **Reversion to normoglycemia**  **(n = 13,605)** | **Persistent prediabetes (n = 18,795)** | **Progression to diabetes (n = 3,125)** | ***P*-value** |
| --- | --- | --- | --- | --- |
| **Systematic inflammation** |  |  |  | <0.001 |
| WBC, 10^9^/L | 6.01 (5.13, 7.04) | 5.79 (4.94, 6.78) | 6.49 (5.54, 7.59) | <0.001 |
| Neutrophil count, 10^9^/L | 3.43 (2.81, 4.22) | 3.33 (2.70, 4.05) | 3.75 (3.06, 4.60) | <0.001 |
| Lymphocyte count, 10^9^/L | 2.00 (1.65, 2.41) | 1.94 (1.61, 2.31) | 2.13 (1.78, 2.57) | <0.001 |
| Monocyte count, 10^9^/L | 0.33 (0.26, 0.42) | 0.32 (0.25, 0.40) | 0.34 (0.27, 0.44) | <0.001 |
| **Oxidative stress** |  |  |  |  |
| GGT, U/L | 21.71 (14.64, 34.39) | 16.00 (11.49, 24.70) | 29.38 (19.67, 47.86) | <0.001 |
| Uric acid, μmol/L | 371.02 (309.00, 435.70) | 331.40 (273.08, 397.70) | 393.78 (333.58, 454.53) | <0.001 |

**Abbreviations:** WBC, white blood cell; GGT, gamma-glutamyl transpeptidase

**Supplemental Table 3** Schoenfeld Residuals Test for PH Assumption.

| **Variable** | **Progression to Diabetes** | | | | | | | |  | **Reversion to Normoglycemia** | | | | | | | |
| --- | --- | --- | --- | --- | --- | --- | --- | --- | --- | --- | --- | --- | --- | --- | --- | --- | --- |
|  | **Model 1** | |  | **Model 3** | |  | **Model 3** | |  | **Model 1** | |  | **Model 2** | |  | **Model 3** | |
|  | ***ρ*** | ***P* value** |  | ***ρ*** | ***P* value** |  | ***ρ*** | ***P* value** |  | ***ρ*** | ***P* value** |  | ***ρ*** | ***P* value** |  | ***ρ*** | ***P* value** |
| **BMI** | 0.501 | 0.479 |  | 1.304 | 0.254 |  | 1.098 | 0.295 |  | 0.338 | 0.561 |  | 0.966 | 0.326 |  | 1.483 | 0.223 |
| **CMI** | 0.561 | 0.454 |  | 0.861 | 0.353 |  | 1.462 | 0.227 |  | 0.470 | 0.493 |  | 0.136 | 0.712 |  | 0.117 | 0.732 |
| **AIP** | 0.401 | 0.527 |  | 0.659 | 0.417 |  | 1.159 | 0.282 |  | 0.223 | 0.637 |  | 0.072 | 0.788 |  | 0.047 | 0.829 |
| **LAP** | 0.974 | 0.324 |  | 1.480 | 0.224 |  | 1.783 | 0.182 |  | 2.251 | 0.134 |  | 1.021 | 0.312 |  | 0.974 | 0.324 |
| **WWI** | 0.046 | 0.829 |  | 0.145 | 0.703 |  | 0.312 | 0.576 |  | 3.129 | 0.077 |  | 3.065 | 0.080 |  | 3.436 | 0.064 |
| **VAI** | 0.200 | 0.655 |  | 0.323 | 0.570 |  | 0.841 | 0.359 |  | 0.475 | 0.491 |  | 0.198 | 0.656 |  | 0.175 | 0.675 |
| **TyG** | 0.010 | 0.920 |  | 0.089 | 0.766 |  | 0.369 | 0.543 |  | 1.280 | 0.258 |  | 0.879 | 0.348 |  | 0.875 | 0.350 |
| **TyG-BMI** | 0.618 | 0.432 |  | 1.473 | 0.225 |  | 1.262 | 0.261 |  | 0.135 | 0.713 |  | 0.540 | 0.463 |  | 0.812 | 0.367 |
| **TyG-WC** | 1.558 | 0.212 |  | 2.466 | 0.116 |  | 1.997 | 0.158 |  | 2.095 | 0.148 |  | 1.366 | 0.242 |  | 1.237 | 0.266 |
| **WHtR** | 0.223 | 0.637 |  | 0.735 | 0.391 |  | 0.740 | 0.390 |  | 1.162 | 0.281 |  | 0.465 | 0.496 |  | 0.323 | 0.570 |
| **TyG-WHtR** | 0.290 | 0.591 |  | 0.652 | 0.420 |  | 0.651 | 0.420 |  | 1.229 | 0.268 |  | 0.665 | 0.415 |  | 0.519 | 0.471 |
| **BRI** | 0.192 | 0.661 |  | 0.683 | 0.409 |  | 0.688 | 0.407 |  | 1.279 | 0.258 |  | 0.498 | 0.480 |  | 0.367 | 0.545 |

**Abbreviations:** *ρ*, Pearson correlation coefficient; BMI, body mass index; CMI, cardiometabolic index; AIP, atherogenic index of plasma; LAP, lipid accumulation index; WWI, weight-adjusted-waist index; VAI, visceral adiposity index; BRI, body roundness index; WHtR, waist circumference/height; TyG, triglyceride-glucose index; TyG-BMI, triglyceride glucose-body mass index; TyG-WC, triglyceride glucose-waist circumference; TyG-WHtR, triglyceride glucose-waist height ratio.

**Supplemental Table 4** Ulticollinearity Test using Variance Inflation Factor.

| **Variable** | **Collinearity Statistics** | |
| --- | --- | --- |
|  | **VIF** | **1/VIF** |
| Age | 1.19 | 0.84 |
| Sex | 1.06 | 0.94 |
| Medication use | 1.23 | 0.81 |
| Smoking | 1.38 | 0.72 |
| Drinking | 1.35 | 0.74 |
| Hypertension | 1.28 | 0.78 |
| Dyslipidemia | 1.04 | 0.96 |

*VIF = Variance Inflation Factor; values <4.0 indicate acceptable multicollinearity.*

**Supplemental Table 5** The Predictive Value of 5-year twelve cardiometabolic indices for the reversion to normoglycemia.

| **Model** | **C-index (95% CI)** | ***P* value** | **NRI (95% CI)** | ***P* value** | **IDI (95% CI)** | ***P* value** |
| --- | --- | --- | --- | --- | --- | --- |
| BMI | 0.645 (0.640-0.650) | < 0.001 | 0.142 (0.038-0.221) | < 0.001 | 0.043 (0.023-0.058) | < 0.001 |
| CMI | 0.625 (0.620-0.630) | < 0.001 | 0.145 (0.075-0.196) | < 0.001 | 0.035 (0.024-0.052) | < 0.001 |
| AIP | 0.608 (0.603-0.613) | < 0.001 | 0.108 (0.041-0.169) | < 0.001 | 0.022 (0.008-0.036) | < 0.001 |
| LAP | 0.647 (0.643-0.652) | < 0.001 | 0.172 (0.090-0.239) | < 0.001 | 0.052 (0.031-0.070) | < 0.001 |
| WWI | 0.604 (0.599-0.609) | < 0.001 | 0.086 (-0.025-0.173) | 0.157 | 0.009 (0.001-0.018) | 0.039 |
| VAI | 0.595 (0.590-0.600) | < 0.001 | 0.107 (0.048-0.166) | < 0.001 | 0.024 (0.013-0.039) | < 0.001 |
| WHtR | 0.657 (0.652-0.662) | < 0.001 | 0.143 (0.037-0.211) | < 0.001 | 0.041 (0.017-0.061) | < 0.001 |
| TyG | 0.617 (0.612-0.622) | < 0.001 | 0.125 (0.041-0.208) | < 0.001 | 0.025 (0.011-0.043) | < 0.001 |
| TyG-BMI | 0.661 (0.656-0.666) | < 0.001 | 0.192 (0.109-0.255) | < 0.001 | 0.054 (0.033-0.073) | < 0.001 |
| TyG-WC | 0.668 (0.663-0.672) | < 0.001 | 0.177 (0.077-0.247) | < 0.001 | 0.054 (0.028-0.073) | < 0.001 |
| TyG-WHtR | 0.673 (0.668-0.677) | < 0.001 | 0.199 (0.099-0.252) | < 0.001 | 0.056 (0.028-0.077) | < 0.001 |
| BRI | 0.657 (0.652-0.662) | < 0.001 | 0.135 (0.041-0.208) | < 0.001 | 0.044 (0.020-0.064) | < 0.001 |

**Abbreviations:** C-index, time-dependent Harrell's concordance indices; NRI, net reclassification improvement; IDI, integrated discrimination improvement; BMI, body mass index; CMI, cardiometabolic index; AIP, atherogenic index of plasma; LAP, lipid accumulation index; WWI, weight-adjusted-waist index; VAI, visceral adiposity index; BRI, body roundness index; WHtR, waist circumference/height; TyG, triglyceride-glucose index; TyG-BMI, triglyceride glucose-body mass index; TyG-WC, triglyceride glucose-waist circumference; TyG-WHtR, triglyceride glucose-waist height ratio.

**Supplemental Table 6** The Predictive Value of 5-year twelve cardiometabolic indices for the progression to diabetes.

| **Model** | **C-index (95% CI)** | ***P* value** | **NRI (95% CI)** | ***P* value** | **IDI (95% CI)** | ***P* value** |
| --- | --- | --- | --- | --- | --- | --- |
| BMI | 0.689 (0.679-0.699) | < 0.001 | 0.246 (0.103-0.412) | < 0.001 | 0.039 (0.011-0.072) | < 0.001 |
| CMI | 0.681 (0.671-0.691) | < 0.001 | 0.136 (0.000-0.290) | 0.039 | 0.026 (0.008-0.045) | < 0.001 |
| AIP | 0.657 (0.647-0.668) | < 0.001 | 0.188 (-0.006-0.301) | 0.078 | 0.025 (0.006-0.040) | < 0.001 |
| LAP | 0.704 (0.695-0.714) | < 0.001 | 0.139 (0.001-0.311) | 0.039 | 0.033 (0.009-0.061) | < 0.001 |
| WWI | 0.658 (0.648-0.668) | < 0.001 | 0.05 (-0.126-0.184) | 0.510 | 0.002 (-0.009-0.015) | 0.627 |
| VAI | 0.649 (0.638-0.660) | < 0.001 | 0.107 (-0.005-0.298) | 0.078 | 0.024 (0.006-0.045) | < 0.001 |
| WHtR | 0.719 (0.710-0.729) | < 0.001 | 0.264 (0.047-0.328) | < 0.001 | 0.036 (0.013-0.056) | < 0.001 |
| TyG | 0.680 (0.670-0.691) | < 0.001 | 0.329 (0.059-0.448) | < 0.001 | 0.055 (0.020-0.092) | < 0.001 |
| TyG-BMI | 0.720 (0.710-0.729) | < 0.001 | 0.241 (0.081-0.414) | < 0.001 | 0.037 (0.010-0.071) | < 0.001 |
| TyG-WC | 0.734 (0.725-0.743) | < 0.001 | 0.224 (0.027-0.347) | < 0.001 | 0.023 (0.002-0.046) | < 0.001 |
| TyG-WHtR | 0.747 (0.739-0.756) | < 0.001 | 0.315 (0.119-0.471) | < 0.001 | 0.039 (0.012-0.068) | < 0.001 |
| BRI | 0.719 (0.710-0.729) | < 0.001 | 0.198 (0.032-0.319) | < 0.001 | 0.023 (0.002-0.046) | < 0.001 |

**Abbreviations:** C-index, time-dependent Harrell's concordance indices; NRI, net reclassification improvement; IDI, integrated discrimination improvement; BMI, body mass index; CMI, cardiometabolic index; AIP, atherogenic index of plasma; LAP, lipid accumulation index; WWI, weight-adjusted-waist index; VAI, visceral adiposity index; BRI, body roundness index; WHtR, waist circumference/height; TyG, triglyceride-glucose index; TyG-BMI, triglyceride glucose-body mass index; TyG-WC, triglyceride glucose-waist circumference; TyG-WHtR, triglyceride glucose-waist height ratio.

**Supplemental Table 7** Associations between TyG-WHtR indices and selected mediators.

| **Mediators** | **Beta** | **Low** | **High** | ***P* value** |
| --- | --- | --- | --- | --- |
| **Systematic inflammation** |  |  |  |  |
| WBC | 0.005 | -0.025 | 0.034 | 0.760 |
| Neutrophil count | 0.056 | 0.025 | 0.086 | <0.001 |
| Lymphocyte count | 0.081 | 0.050 | 0.113 | <0.001 |
| Monocyte count | -0.130 | -0.193 | -0.067 | <0.001 |
| **Oxidative stress**  GGT |  |  |  |  |
|  | 0.013 | 0.012 | 0.013 | <0.001 |
| Uric acid | 0.002 | 0.002 | 0.002 | <0.001 |
| Linear regression models were used to estimate the association between TyG-WHtR indices and selected mediators. Models were adjusted for age, sex, dyslipidemia, hypertension, medication use, drinking and smoking.  **Abbreviations:** WBC, white blood cell; GGT, gamma-glutamyl transpeptidase; TyG-WHtR, triglyceride glucose-waist height ratio. | | | | |

**Supplemental Table 8** Association between selected mediators and risk of progression to diabetes and reversion to normoglycemia.

| **Mediators** | **HR** | **Low** | **High** | ***P* value** |  |
| --- | --- | --- | --- | --- | --- |
| **Progression to diabetes** |  |  |  |  |  |
| **Systematic inflammation** |  |  |  |  |  |
| WBC | 0.833 | 0.701 | 0.990 | 0.038 |  |
| Neutrophil count | 1.449 | 1.210 | 1.730 | <0.001 |  |
| lymphocyte count | 1.386 | 1.150 | 1.670 | <0.001 |  |
| Monocyte count | 1.122 | 0.747 | 1.680 | 0.579 |  |
| **Oxidative stress**  GGT |  |  |  |  |  |
|  | 1.017 | 1.010 | 1.020 | <0.001 |  |
| Uric acid | 1.002 | 1.000 | 1.007 | <0.001 |  |
| **Reversion to normoglycemia** |  |  |  |  |  |
| **Systematic inflammation** |  |  |  |  |  |
| WBC | 1.080 | 0.983 | 1.190 | 0.108 |  |
| Neutrophil count | 0.842 | 0.762 | 0.929 | <0.001 |  |
| lymphocyte count | 0.791 | 0.714 | 0.876 | <0.001 |  |
| Monocyte count | 2.596 | 2.120 | 3.180 | <0.001 |  |
| **Oxidative stress**  GGT |  |  |  |  |  |
|  | 0.987 | 0.985 | 0.988 | <0.001 |  |
| Uric acid | 0.999 | 0.999 | 0.999 | <0.001 |  |
| Cox hazard proportion regression model were used to estimate the association between selected mediators and risk of progression to diabetes and reversion to normoglycemia. Models were adjusted for age, sex, dyslipidemia, hypertension, medication use, drinking and smoking.  **Abbreviation:** WBC, white blood cell; GGT, gamma-glutamyl transpeptidase; HR, hazard ratio. | | | | | |

| **Supplemental Table 9** Mediated role of selected biomarkers in association between TyG-WHtR indices and risk of progression to diabetes and reversion to normoglycemia. | | | | | |
| --- | --- | --- | --- | --- | --- |
| **Mediators** | **Total effect** | **Indirect effect** | **Direct effect** | **Mediated proportion (%)** | ***P* value** |
| **Progression to diabetes** |  |  |  |  |  |
| **Systematic inflammation** |  |  |  |  |  |
| Neutrophill count | 2.3038 (1.7400, 2.9466)^a^ | 8.9320 (5.8284, 13.1370)^b^ | 2.2145 (1.6793, 2.8285)^a^ | 3.88 (2.86, 4.95) | <0.001 |
| Lymphocyte count | 2.3285 (1.7483, 2.9859)^a^ | 7.5530 (4.5390, 10.7210)^b^ | 2.2529 (1.6903, 2.9033)^a^ | 3.24 (2.25, 4.31) | <0.001 |
| **Oxidative stress**  GGT |  |  |  |  |  |
|  | 2.3295 (1.7487, 2.9860)^a^ | 19.2330 (12.6100, 25.9480)^b^ | 2.1372 (1.6209, 2.7374)^a^ | 8.26 (6.51, 9.77) | <0.001 |
| **Reversion to normoglycemia** |  |  |  |  |  |
| **Systematic inflammation** |  |  |  |  |  |
| Neutrophill count | -0.0115 (-0.0135, -0.0097) | -0.9120 (-1.7596, -0.1302)^b^ | -0.0114 (-0.0134, -0.0096) | 0.80 (0.11, 1.46) | <0.05 |
| **Oxidative stress**  GGT |  |  |  |  |  |
|  | -0.0115 (-0.0135, -0.0097) | -0.0005 (-0.0006, -0.0003) | -0.0110 (-0.0129, -0.0093) | 4.05 (2.85, 5.26) | <0.001 |
| Uric acid | -0.0115 (-0.0136, -0.0097) | -0.0003 (-0.0005, -0.0002) | -0.0111 (-0.0131, -0.0094) | 2.90 (1.78, 4.03) | <0.001 |
| Models were adjusted for age, sex, dyslipidemia, hypertension, medication use, drinking and smoking. **Abbreviations:** GGT, gamma-glutamyl transpeptidase; TyG-WHtR, triglyceride glucose-waist height ratio. | | | | | |
| ^a^ indicates the displayed value multiplied by×10^-4^  ^b^ indicates the displayed value multiplied by×10^-6^ | | | | | |

**Supplemental Table 10** Association between twelve cardiometabolic indices and the progression to diabetes after excluding individuals with FPG <6.1 mmol/L according to WHO recommended criteria.

| **Variable** | **Model 1** | | | | **Model 2** | | | | | **Model 3** | | |
| --- | --- | --- | --- | --- | --- | --- | --- | --- | --- | --- | --- | --- |
|  | **HR** | **95% CI** | ***P*-value** | | **HR** | | **95% CI** | | ***P*-value** | **HR** | **95% CI** | ***P*-value** |
| BMI | 2.13 | (2.00, 2.26) | <0.001 | 2.13 | | (2.00, 2.26) | | <0.001 | | 2.13 | (2.00, 2.26) | <0.001 |
| CMI | 1.59 | (1.51, 1.67) | <0.001 | 1.59 | | (1.51, 1.67) | | <0.001 | | 1.59 | (1.51, 1.67) | <0.001 |
| AIP | 1.65 | (1.55, 1.76) | <0.001 | 1.65 | | (1.55, 1.76) | | <0.001 | | 1.65 | (1.55, 1.76) | <0.001 |
| LAP | 1.72 | (1.64, 1.81) | <0.001 | 1.72 | | (1.64, 1.81) | | <0.001 | | 1.72 | (1.64, 1.81) | <0.001 |
| WWI | 1.64 | (1.54, 1.75) | <0.001 | 1.64 | | (1.54, 1.75) | | <0.001 | | 1.64 | (1.54, 1.75) | <0.001 |
| VAI | 1.50 | (1.43, 1.58) | <0.001 | 1.50 | | (1.43, 1.58) | | <0.001 | | 1.50 | (1.43, 1.58) | <0.001 |
| WHtR | 2.21 | (2.07, 2.35) | <0.001 | 2.21 | | (2.07, 2.35) | | <0.001 | | 2.21 | (2.07, 2.35) | <0.001 |
| TyG | 1.64 | (1.53, 1.75) | <0.001 | 1.64 | | (1.53, 1.75) | | <0.001 | | 1.64 | (1.53, 1.75) | <0.001 |
| TyG-BMI | 2.18 | (2.05, 2.32) | <0.001 | 2.18 | | (2.05, 2.32) | | <0.001 | | 2.18 | (2.05, 2.32) | <0.001 |
| TyG-WC | 2.19 | (2.06, 2.33) | <0.001 | 2.19 | | (2.06, 2.33) | | <0.001 | | 2.19 | (2.06, 2.33) | <0.001 |
| TyG-WHtR | 2.30 | (2.16, 2.45) | <0.001 | 2.30 | | (2.16, 2.45) | | <0.001 | | 2.30 | (2.16, 2.45) | <0.001 |
| BRI | 2.09 | (1.97, 2.21) | <0.001 | 2.09 | | (1.97, 2.21) | | <0.001 | | 2.09 | (1.97, 2.21) | <0.001 |

**Model 1:** Unadjusted

**Model 2:** Adjusted for age and sex

**Model 3:** Adjusted for age, sex, dyslipidemia, hypertension, medication use, drinking and smoking

**Abbreviations:** HR, Hazard ratio; CI, Confidence interval; FPG, fasting plasma glucose; BMI, body mass index; CMI, cardiometabolic index; AIP, atherogenic index of plasma; LAP, lipid accumulation index; WWI, weight-adjusted-waist index; VAI, visceral adiposity index; BRI, body roundness index; WHtR, waist circumference/height; TyG, triglyceride-glucose index; TyG-BMI, triglyceride glucose-body mass index; TyG-WC, triglyceride glucose-waist circumference; TyG-WHtR, triglyceride glucose-waist height ratio.

**Supplemental Table 11** Association between twelve cardiometabolic indices and the reversion to normoglycemia after excluding individuals with FPG <6.1 mmol/L according to WHO recommended criteria.

| **Variable** | **Model 1** | | | | **Model 2** | | | | | **Model 3** | | |
| --- | --- | --- | --- | --- | --- | --- | --- | --- | --- | --- | --- | --- |
|  | **HR** | **95% CI** | ***P*-value** | | **HR** | | **95% CI** | | ***P*-value** | **HR** | **95% CI** | ***P*-value** |
| BMI | 0.64 | (0.63, 0.65) | <0.001 | 0.64 | | (0.63, 0.65) | | <0.001 | | 0.64 | (0.63, 0.65) | <0.001 |
| CMI | 0.63 | (0.62, 0.64) | <0.001 | 0.63 | | (0.62, 0.64) | | <0.001 | | 0.63 | (0.62, 0.64) | <0.001 |
| AIP | 0.71 | (0.70, 0.72) | <0.001 | 0.71 | | (0.70, 0.72) | | <0.001 | | 0.71 | (0.70, 0.72) | <0.001 |
| LAP | 0.57 | (0.56, 0.58) | <0.001 | 0.57 | | (0.56, 0.58) | | <0.001 | | 0.57 | (0.56, 0.58) | <0.001 |
| WWI | 0.75 | (0.74, 0.77) | <0.001 | 0.75 | | (0.74, 0.77) | | <0.001 | | 0.75 | (0.74, 0.77) | <0.001 |
| VAI | 0.71 | (0.70, 0.73) | <0.001 | 0.71 | | (0.70, 0.73) | | <0.001 | | 0.71 | (0.70, 0.73) | <0.001 |
| WHtR | 0.62 | (0.61, 0.63) | <0.001 | 0.62 | | (0.61, 0.63) | | <0.001 | | 0.62 | (0.61, 0.63) | <0.001 |
| TyG | 0.71 | (0.70, 0.73) | <0.001 | 0.71 | | (0.70, 0.73) | | <0.001 | | 0.71 | (0.70, 0.73) | <0.001 |
| TyG-BMI | 0.60 | (0.59, 0.61) | <0.001 | 0.60 | | (0.59, 0.61) | | <0.001 | | 0.60 | (0.59, 0.61) | <0.001 |
| TyG-WC | 0.59 | (0.58, 0.61) | <0.001 | 0.59 | | (0.58, 0.61) | | <0.001 | | 0.59 | (0.58, 0.61) | <0.001 |
| TyG-WHtR | 0.59 | (0.58, 0.60) | <0.001 | 0.59 | | (0.58, 0.60) | | <0.001 | | 0.59 | (0.58, 0.60) | <0.001 |
| BRI | 0.60 | (0.59, 0.61) | <0.001 | 0.60 | | (0.59, 0.61) | | <0.001 | | 0.60 | (0.59, 0.61) | <0.001 |

**Model 1:** Unadjusted

**Model 2:** Adjusted for age and sex

**Model 3:** Adjusted for age, sex, dyslipidemia, hypertension, medication use, drinking and smoking

**Abbreviations:** HR, Hazard ratio; CI, Confidence interval; FPG, fasting plasma glucose; BMI, body mass index; CMI, cardiometabolic index; AIP, atherogenic index of plasma; LAP, lipid accumulation index; WWI, weight-adjusted-waist index; VAI, visceral adiposity index; BRI, body roundness index; WHtR, waist circumference/height; TyG, triglyceride-glucose index; TyG-BMI, triglyceride glucose-body mass index; TyG-WC, triglyceride glucose-waist circumference; TyG-WHtR, triglyceride glucose-waist height ratio.

**Supplemental Table 12** Association between twelve cardiometabolic indices and the progression to diabetes after excluding individuals who were lost to follow-up or with follow-up duration <2 years.

| **Variable** | **Model 1** | | | | **Model 2** | | | | | **Model 3** | | |
| --- | --- | --- | --- | --- | --- | --- | --- | --- | --- | --- | --- | --- |
|  | **HR** | **95% CI** | ***P*-value** | | **HR** | | **95% CI** | | ***P*-value** | **HR** | **95% CI** | ***P*-value** |
| BMI | 1.99 | (1.91, 2.07) | <0.001 | 2.00 | | (1.91, 2.08) | | <0.001 | | 1.90 | (1.81, 1.98) | <0.001 |
| CMI | 1.64 | (1.59, 1.70) | <0.001 | 1.63 | | (1.57, 1.69) | | <0.001 | | 1.72 | (1.64, 1.81) | <0.001 |
| AIP | 1.70 | (1.63, 1.77) | <0.001 | 1.70 | | (1.63, 1.77) | | <0.001 | | 1.70 | (1.61, 1.80) | <0.001 |
| LAP | 1.79 | (1.73, 1.85) | <0.001 | 1.74 | | (1.69, 1.81) | | <0.001 | | 1.81 | (1.73, 1.89) | <0.001 |
| WWI | 1.65 | (1.58, 1.71) | <0.001 | 1.53 | | (1.46, 1.60) | | <0.001 | | 1.47 | (1.40, 1.53) | <0.001 |
| VAI | 1.55 | (1.49, 1.60) | <0.001 | 1.55 | | (1.50, 1.61) | | <0.001 | | 1.57 | (1.49, 1.65) | <0.001 |
| WHtR | 2.14 | (2.06, 2.23) | <0.001 | 2.02 | | (1.94, 2.11) | | <0.001 | | 1.93 | (1.85, 2.01) | <0.001 |
| TyG | 1.91 | (1.83, 1.99) | <0.001 | 1.85 | | (1.77, 1.93) | | <0.001 | | 1.96 | (1.85, 2.08) | <0.001 |
| TyG-BMI | 2.17 | (2.09, 2.26) | <0.001 | 2.21 | | (2.12, 2.30) | | <0.001 | | 2.14 | (2.04, 2.24) | <0.001 |
| TyG-WC | 2.26 | (2.17, 2.35) | <0.001 | 2.34 | | (2.24, 2.45) | | <0.001 | | 2.31 | (2.20, 2.42) | <0.001 |
| TyG-WHtR | 2.38 | (2.29, 2.48) | <0.001 | 2.27 | | (2.18, 2.37) | | <0.001 | | 2.25 | (2.15, 2.36) | <0.001 |
| BRI | 2.05 | (1.97, 2.12) | <0.001 | 1.93 | | (1.86, 2.01) | | <0.001 | | 1.85 | (1.78, 1.93) | <0.001 |

**Model 1:** Unadjusted

**Model 2:** Adjusted for age and sex

**Model 3:** Adjusted for age, sex, dyslipidemia, hypertension, medication use, drinking and smoking

**Abbreviations:** HR, Hazard ratio; CI, Confidence interval; FPG, fasting plasma glucose; BMI, body mass index; CMI, cardiometabolic index; AIP, atherogenic index of plasma; LAP, lipid accumulation index; WWI, weight-adjusted-waist index; VAI, visceral adiposity index; BRI, body roundness index; WHtR, waist circumference/height; TyG, triglyceride-glucose index; TyG-BMI, triglyceride glucose-body mass index; TyG-WC, triglyceride glucose-waist circumference; TyG-WHtR, triglyceride glucose-waist height ratio.

**Supplemental Table 13** Association between twelve cardiometabolic indices and the reversion to normoglycemia after excluding individuals who were lost to follow-up or with follow-up duration <2 years.

| **Variable** | **Model 1** | | | | **Model 2** | | | | | **Model 3** | | |
| --- | --- | --- | --- | --- | --- | --- | --- | --- | --- | --- | --- | --- |
|  | **HR** | **95% CI** | ***P*-value** | | **HR** | | **95% CI** | | ***P*-value** | **HR** | **95% CI** | ***P*-value** |
| BMI | 0.62 | (0.60, 0.63) | <0.001 | 0.67 | | (0.66, 0.68) | | <0.001 | | 0.69 | (0.68, 0.70) | <0.001 |
| CMI | 0.60 | (0.59, 0.61) | <0.001 | 0.65 | | (0.64, 0.67) | | <0.001 | | 0.64 | (0.62, 0.66) | <0.001 |
| AIP | 0.69 | (0.68, 0.70) | <0.001 | 0.74 | | (0.72, 0.75) | | <0.001 | | 0.76 | (0.74, 0.78) | <0.001 |
| LAP | 0.54 | (0.53, 0.55) | <0.001 | 0.60 | | (0.58, 0.61) | | <0.001 | | 0.59 | (0.57, 0.61) | <0.001 |
| WWI | 0.73 | (0.71, 0.74) | <0.001 | 0.82 | | (0.81, 0.84) | | <0.001 | | 0.85 | (0.83, 0.86) | <0.001 |
| VAI | 0.69 | (0.68, 0.71) | <0.001 | 0.71 | | (0.70, 0.73) | | <0.001 | | 0.72 | (0.70, 0.74) | <0.001 |
| WHtR | 0.59 | (0.58, 0.60) | <0.001 | 0.66 | | (0.65, 0.67) | | <0.001 | | 0.69 | (0.67, 0.70) | <0.001 |
| TyG | 0.67 | (0.66, 0.68) | <0.001 | 0.73 | | (0.72, 0.74) | | <0.001 | | 0.75 | (0.74, 0.77) | <0.001 |
| TyG-BMI | 0.57 | (0.56, 0.58) | <0.001 | 0.62 | | (0.61, 0.63) | | <0.001 | | 0.64 | (0.62, 0.65) | <0.001 |
| TyG-WC | 0.56 | (0.54, 0.57) | <0.001 | 0.59 | | (0.58, 0.60) | | <0.001 | | 0.60 | (0.59, 0.62) | <0.001 |
| TyG-WHtR | 0.55 | (0.54, 0.56) | <0.001 | 0.61 | | (0.60, 0.62) | | <0.001 | | 0.62 | (0.61, 0.64) | <0.001 |
| BRI | 0.57 | (0.56, 0.58) | <0.001 | 0.64 | | (0.63, 0.65) | | <0.001 | | 0.67 | (0.65, 0.68) | <0.001 |

**Model 1:** Unadjusted

**Model 2:** Adjusted for age and sex

**Model 3:** Adjusted for age, sex, dyslipidemia, hypertension, medication use, drinking and smoking

**Abbreviations:** HR, Hazard ratio; CI, Confidence interval; FPG, fasting plasma glucose; BMI, body mass index; CMI, cardiometabolic index; AIP, atherogenic index of plasma; LAP, lipid accumulation index; WWI, weight-adjusted-waist index; VAI, visceral adiposity index; BRI, body roundness index; WHtR, waist circumference/height; TyG, triglyceride-glucose index; TyG-BMI, triglyceride glucose-body mass index; TyG-WC, triglyceride glucose-waist circumference; TyG-WHtR, triglyceride glucose-waist height ratio.

**Supplemental Table 14** Association between twelve cardiometabolic indices and the progression to diabetes after excluding individuals with any self-reported current medication use at baseline.

| **Variable** | **Model 1** | | | | **Model 2** | | | | | **Model 3** | | |
| --- | --- | --- | --- | --- | --- | --- | --- | --- | --- | --- | --- | --- |
|  | **HR** | **95% CI** | ***P*-value** | | **HR** | | **95% CI** | | ***P*-value** | **HR** | **95% CI** | ***P*-value** |
| BMI | 1.89 | (1.80, 1.98) | <0.001 | 1.87 | | (1.78, 1.97) | | <0.001 | | 1.77 | (1.68, 1.86) | <0.001 |
| CMI | 1.55 | (1.48, 1.61) | <0.001 | 1.54 | | (1.48, 1.61) | | <0.001 | | 1.62 | (1.52, 1.72) | <0.001 |
| AIP | 1.55 | (1.48, 1.62) | <0.001 | 1.56 | | (1.48, 1.64) | | <0.001 | | 1.55 | (1.46, 1.66) | <0.001 |
| LAP | 1.70 | (1.63, 1.77) | <0.001 | 1.67 | | (1.60, 1.74) | | <0.001 | | 1.72 | (1.63, 1.82) | <0.001 |
| WWI | 1.54 | (1.47, 1.61) | <0.001 | 1.47 | | (1.39, 1.54) | | <0.001 | | 1.40 | (1.33, 1.48) | <0.001 |
| VAI | 1.45 | (1.40, 1.52) | <0.001 | 1.47 | | (1.41, 1.53) | | <0.001 | | 1.48 | (1.39, 1.56) | <0.001 |
| WHtR | 1.97 | (1.88, 2.06) | <0.001 | 1.88 | | (1.79, 1.98) | | <0.001 | | 1.79 | (1.70, 1.88) | <0.001 |
| TyG | 1.70 | (1.61, 1.78) | <0.001 | 1.66 | | (1.58, 1.75) | | <0.001 | | 1.73 | (1.61, 1.85) | <0.001 |
| TyG-BMI | 2.02 | (1.93, 2.12) | <0.001 | 2.03 | | (1.94, 2.14) | | <0.001 | | 1.96 | (1.85, 2.07) | <0.001 |
| TyG-WC | 2.07 | (1.97, 2.17) | <0.001 | 2.12 | | (2.02, 2.24) | | <0.001 | | 2.08 | (1.96, 2.20) | <0.001 |
| TyG-WHtR | 2.14 | (2.04, 2.25) | <0.001 | 2.06 | | (1.96, 2.17) | | <0.001 | | 2.03 | (1.92, 2.14) | <0.001 |
| BRI | 1.90 | (1.82, 1.99) | <0.001 | 1.83 | | (1.74, 1.91) | | <0.001 | | 1.73 | (1.65, 1.82) | <0.001 |

**Model 1:** Unadjusted

**Model 2:** Adjusted for age and sex

**Model 3:** Adjusted for age, sex, dyslipidemia, hypertension, medication use, drinking and smoking

**Abbreviations:** HR, Hazard ratio; CI, Confidence interval; FPG, fasting plasma glucose; BMI, body mass index; CMI, cardiometabolic index; AIP, atherogenic index of plasma; LAP, lipid accumulation index; WWI, weight-adjusted-waist index; VAI, visceral adiposity index; BRI, body roundness index; WHtR, waist circumference/height; TyG, triglyceride-glucose index; TyG-BMI, triglyceride glucose-body mass index; TyG-WC, triglyceride glucose-waist circumference; TyG-WHtR, triglyceride glucose-waist height ratio.

**Supplemental Table 15** Association between twelve cardiometabolic indices and the reversion to normoglycemia after excluding individuals with any self-reported current medication use at baseline.

| **Variable** | **Model 1** | | | | **Model 2** | | | | | **Model 3** | | |
| --- | --- | --- | --- | --- | --- | --- | --- | --- | --- | --- | --- | --- |
|  | **HR** | **95% CI** | ***P*-value** | | **HR** | | **95% CI** | | ***P*-value** | **HR** | **95% CI** | ***P*-value** |
| BMI | 0.57 | (0.55, 0.58) | <0.001 | 0.62 | | (0.60, 0.64) | | <0.001 | | 0.64 | (0.62, 0.66) | <0.001 |
| CMI | 0.58 | (0.55, 0.60) | <0.001 | 0.62 | | (0.60, 0.65) | | <0.001 | | 0.61 | (0.58, 0.64) | <0.001 |
| AIP | 0.67 | (0.65, 0.69) | <0.001 | 0.71 | | (0.69, 0.74) | | <0.001 | | 0.74 | (0.71, 0.76) | <0.001 |
| LAP | 0.51 | (0.49, 0.53) | <0.001 | 0.57 | | (0.54, 0.59) | | <0.001 | | 0.56 | (0.53, 0.59) | <0.001 |
| WWI | 0.71 | (0.69, 0.73) | <0.001 | 0.82 | | (0.79, 0.84) | | <0.001 | | 0.84 | (0.82, 0.87) | <0.001 |
| VAI | 0.67 | (0.65, 0.70) | <0.001 | 0.69 | | (0.66, 0.71) | | <0.001 | | 0.69 | (0.66, 0.73) | <0.001 |
| WHtR | 0.56 | (0.55, 0.58) | <0.001 | 0.64 | | (0.61, 0.66) | | <0.001 | | 0.66 | (0.64, 0.69) | <0.001 |
| TyG | 0.65 | (0.64, 0.67) | <0.001 | 0.71 | | (0.69, 0.73) | | <0.001 | | 0.73 | (0.70, 0.75) | <0.001 |
| TyG-BMI | 0.52 | (0.51, 0.54) | <0.001 | 0.57 | | (0.55, 0.59) | | <0.001 | | 0.59 | (0.57, 0.61) | <0.001 |
| TyG-WC | 0.53 | (0.52, 0.55) | <0.001 | 0.57 | | (0.55, 0.59) | | <0.001 | | 0.59 | (0.57, 0.61) | <0.001 |
| TyG-WHtR | 0.53 | (0.51, 0.54) | <0.001 | 0.59 | | (0.57, 0.61) | | <0.001 | | 0.60 | (0.58, 0.62) | <0.001 |
| BRI | 0.54 | (0.52, 0.56) | <0.001 | 0.61 | | (0.59, 0.64) | | <0.001 | | 0.64 | (0.62, 0.67) | <0.001 |

**Model 1:** Unadjusted

**Model 2:** Adjusted for age and sex

**Model 3:** Adjusted for age, sex, dyslipidemia, hypertension, medication use, drinking and smoking

**Abbreviations:** HR, Hazard ratio; CI, Confidence interval; FPG, fasting plasma glucose; BMI, body mass index; CMI, cardiometabolic index; AIP, atherogenic index of plasma; LAP, lipid accumulation index; WWI, weight-adjusted-waist index; VAI, visceral adiposity index; BRI, body roundness index; WHtR, waist circumference/height; TyG, triglyceride-glucose index; TyG-BMI, triglyceride glucose-body mass index; TyG-WC, triglyceride glucose-waist circumference; TyG-WHtR, triglyceride glucose-waist height ratio.

**Supplemental Figure 1.** Flow chart of study subjects.

**
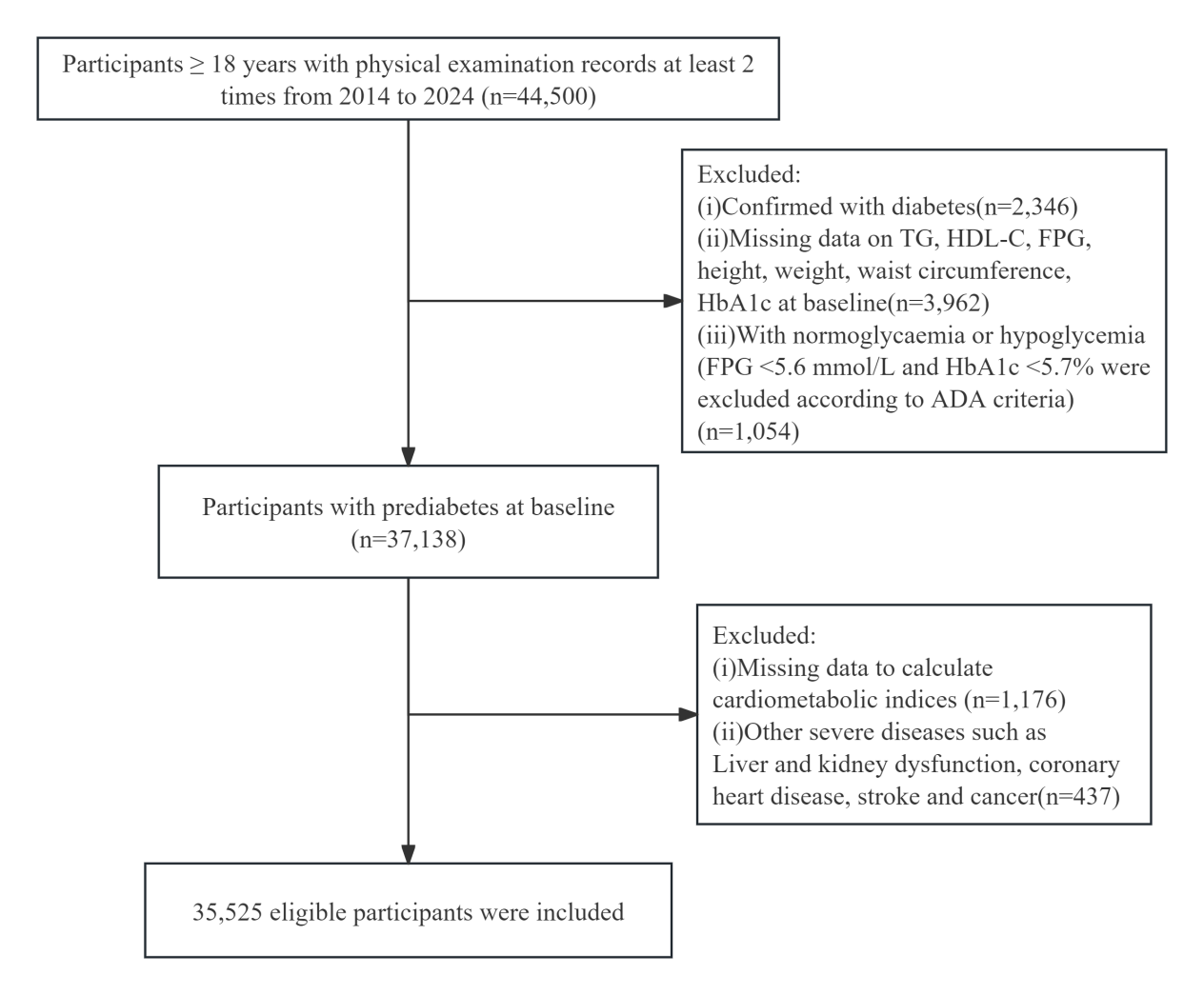
**

**Supplemental Figure 2.** Summary of missing variables.

The missing proportion of WC, weight, height, BMI and Hb was 12.2%, 4.3%, 4.3%, 2.4% and 0.5%, respectively.

**Abbreviations:** SBP, systolic blood pressure; DBP, diastolic blood pressure; WC, waist circumference; Hb, hemoglobin; FPG, fasting plasma glucose; TC, total cholesterol; TG, triglyceride; HDL-C, high-density lipoprotein cholesterol; LDL-C, low-density lipoprotein cholesterol; BMI, body mass index.

**
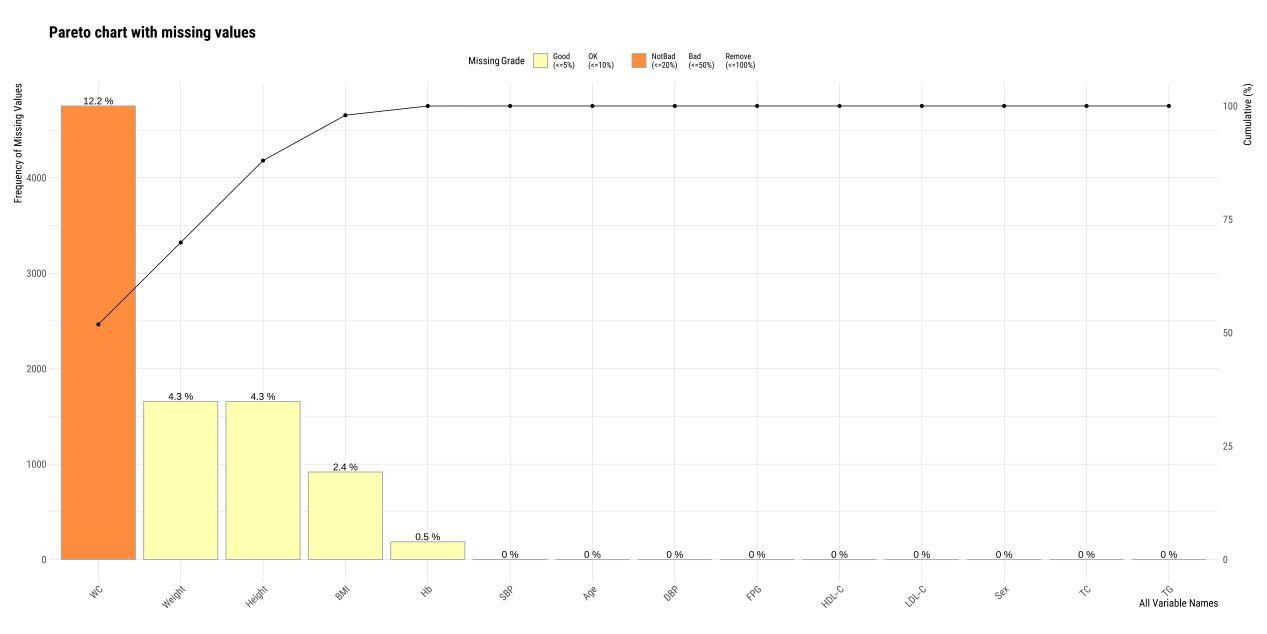
**

**Supplemental Figure 3.** The final constructed directed acyclic graph.

The green node at the bottom is the exposure variable (cardiometabolic indices), and the blue node with I at the bottom right is the outcome variable (diabetes). The remaining pink and blue nodes represent observed confounders, where the blue nodes are the ancestor of the outcome. The directed edges represent the causal relationship between each variable pair, which starts from the cause and ends at the outcome. The green arrow represents the causal path, including the direct causal path from exposure to the outcome, and the indirect causal path through the mediators (dyslipidemia). Mediators are defined as variables that are caused by the exposure and in turn cause the outcome. The pink arrows represent the biasing path.


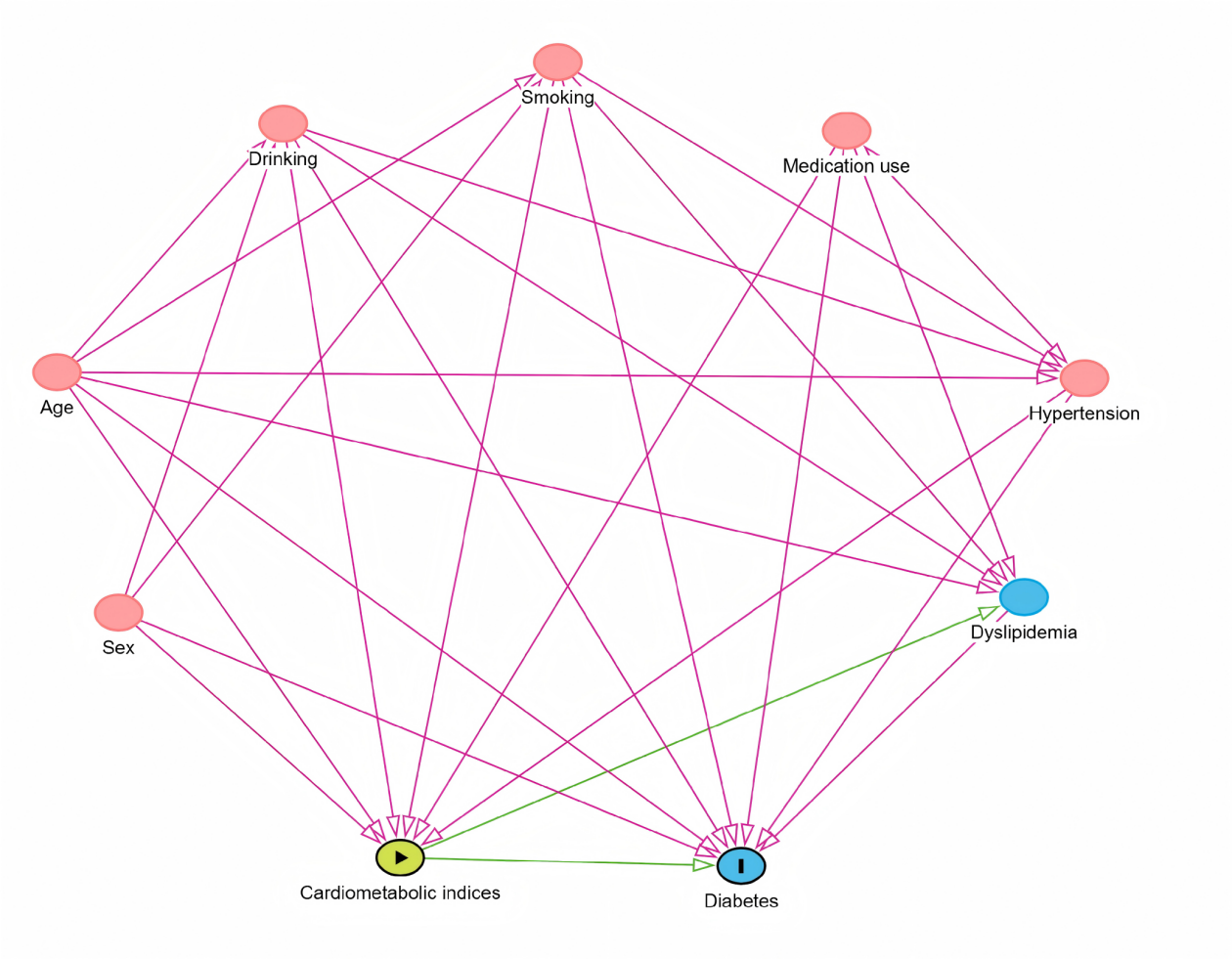


**Supplemental Figure 4.** The Predictive Value of 5-year twelve cardiometabolic indices for the progression to diabetes as well as reversion to normoglycemia.

**Abbreviations:** BMI, body mass index; CMI, cardiometabolic index; AIP, atherogenic index of plasma; LAP, lipid accumulation index; WWI, weight-adjusted-waist index; VAI, visceral adiposity index; BRI, body roundness index; WHtR, waist circumference/height; TyG, triglyceride-glucose index; TyG-BMI, triglyceride glucose-body mass index; TyG-WC, triglyceride glucose-waist circumference; TyG-WHtR, triglyceride glucose-waist height ratio.


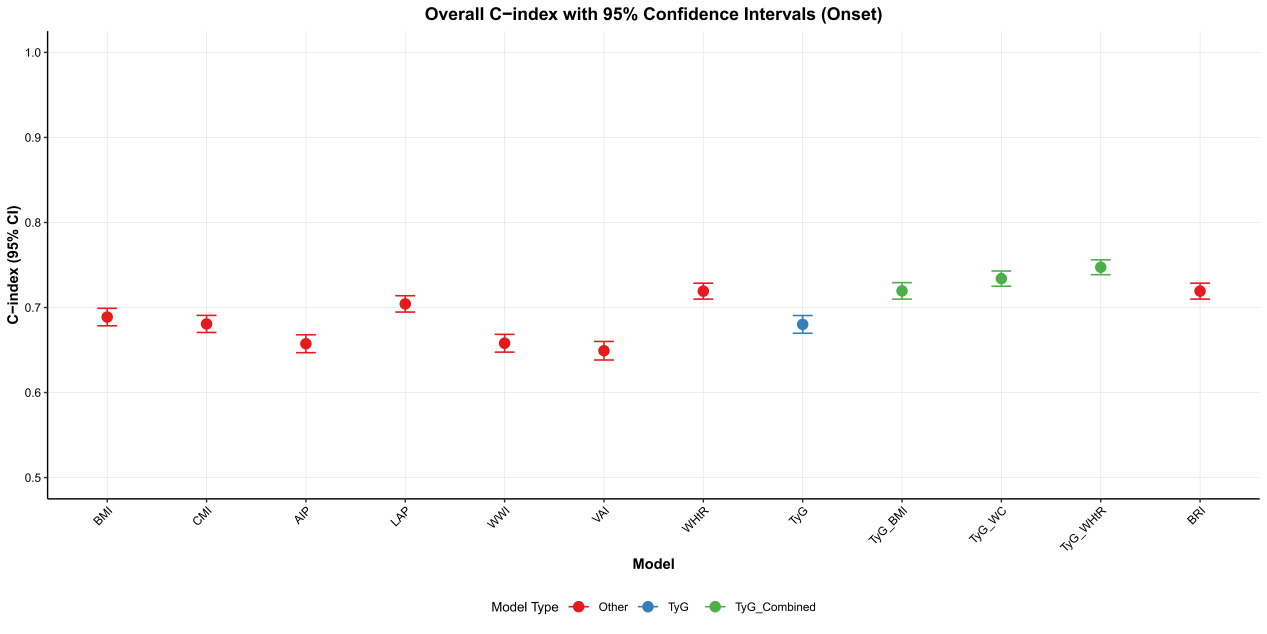


**Supplemental Figure 5.** The Predictive Value of 5-year twelve cardiometabolic indices for the reversion to normoglycemia.

**Abbreviations:** BMI, body mass index; CMI, cardiometabolic index; AIP, atherogenic index of plasma; LAP, lipid accumulation index; WWI, weight-adjusted-waist index; VAI, visceral adiposity index; BRI, body roundness index; WHtR, waist circumference/height; TyG, triglyceride-glucose index; TyG-BMI, triglyceride glucose-body mass index; TyG-WC, triglyceride glucose-waist circumference; TyG-WHtR, triglyceride glucose-waist height ratio.


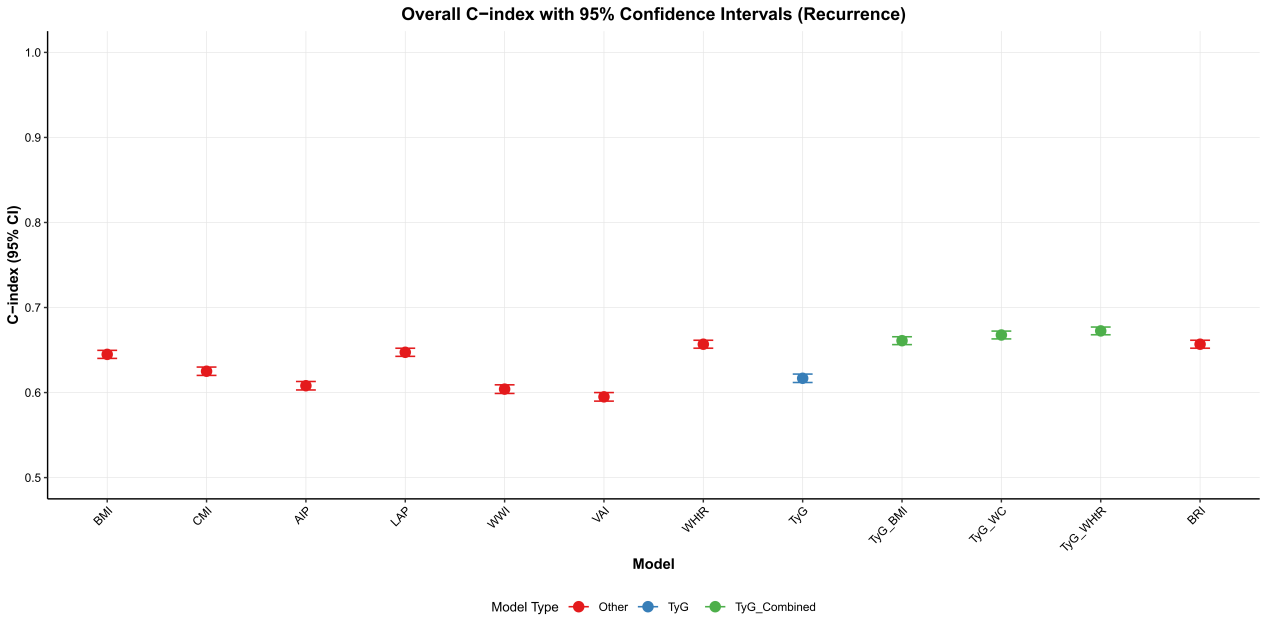


**Supplemental Figure 6.** Subgroup analyses of the association of TyG-WHtR with progression to diabetes (A) and reversion to normoglycemia (B).

**Abbreviations:** TyG-WHtR, triglyceride glucose-waist height ratio; HR, Hazard ratio; CI, Confidence interval; BMI, body mass index; FPG, fasting plasma glucose.


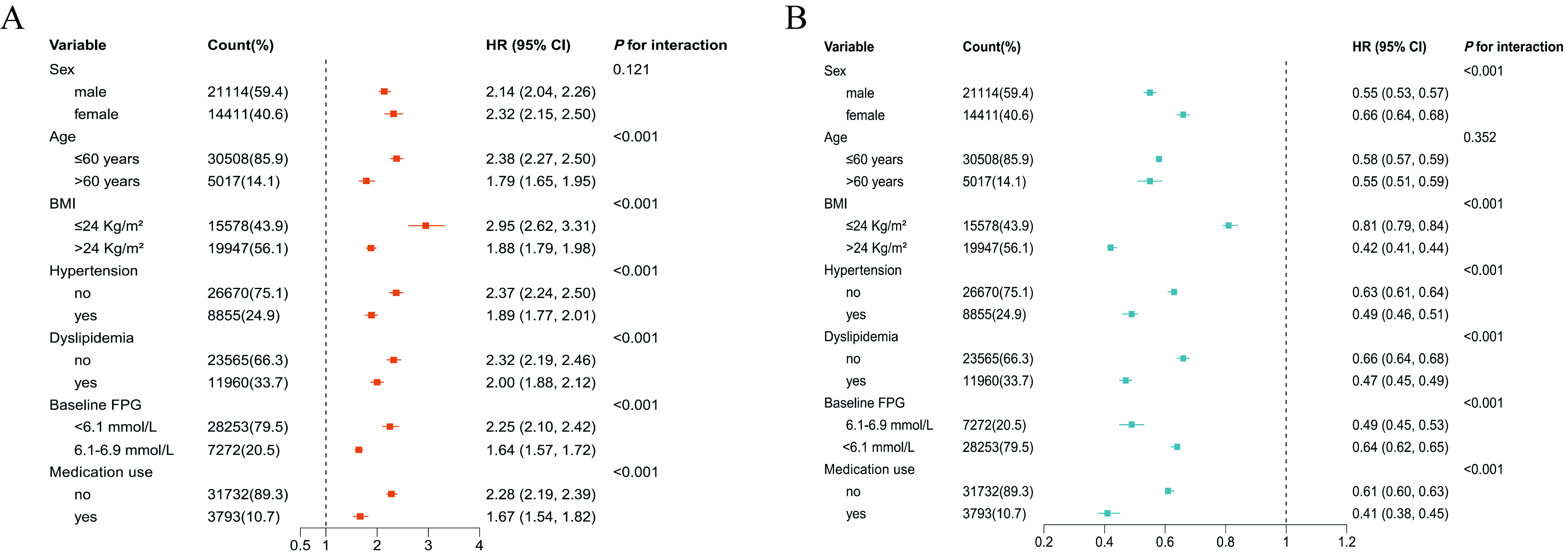

Supplement: Supplementary file 1 [file Supplementary_file_1.docx]
